# Immunogenicity and Safety of the ExPEC9V *Escherichia coli* Vaccine Co-Administered with a High-Dose Influenza Vaccine in Older Adults: A Placebo-Controlled, Randomized, Phase 3 Study

**DOI:** 10.3390/vaccines14020146

**Published:** 2026-01-30

**Authors:** Isabel Leroux-Roels, Tracey A. Day, Sofie Deleu, Chelsea McLean, Oscar Go, Todd A. Davies, Jeroen N. Stoop, Monika Peeters, Maria G. Pau, Bart Spiessens, Michal Sarnecki, Keira A. Cohen

**Affiliations:** 1 Center for Vaccinology, Ghent University and Ghent University Hospital, Corneel Heymanslaan 10, 9000 Ghent, Belgium; isabel.lerouxroels@uzgent.be; 2 Johnson & Johnson, Turnhoutseweg 30, 2340 Beerse, Belgium; mpeeter7@its.jnj.com (M.P.); bartspiessens0@gmail.com (B.S.); 3 Johnson & Johnson, Archimedesweg 4, 2333CN Leiden, The Netherlands; cmclean@its.jnj.com (C.M.); jstoop1@its.jnj.com (J.N.S.); mpau@its.jnj.com (M.G.P.); 4 Johnson & Johnson, 920 US-202, Raritan, NJ 08869, USA; ogo@its.jnj.com (O.G.); tdavies1@its.jnj.com (T.A.D.); 5 Johnson & Johnson, Rehhagstrasse 79, 3018 Bern, Switzerland; 6 Johnson & Johnson, 1125 Bear Tavern Rd, New Brunswick, NJ 08560, USA

**Keywords:** clinical trial, *Escherichia coli*, ExPEC9V, high-dose influenza vaccine, invasive *E. coli* disease, O-serotypes, vaccine, vaccine co-administration

## Abstract

Background: ExPEC9V is a 9-valent vaccine candidate designed to prevent invasive *Escherichia coli* disease, a life-threatening condition occurring when extraintestinal pathogenic *E. coli* (ExPEC) invade sterile sites. We evaluated immunogenicity and safety when ExPEC9V was co-administered with high-dose (HD) quadrivalent seasonal influenza vaccine. Methods: This Phase 3, double-blind, placebo-controlled study (NCT06134804) randomized 959 adults (≥65 years) to receive co-administration of ExPEC9V and HD quadrivalent seasonal influenza vaccine (CoAd) or each vaccine alone, 29 days apart (Control). Co-primary objectives were non-inferiority of co-administration versus separate administration following predefined criteria based on influenza strain-specific hemagglutination inhibition (HAI) antibody titers and ExPEC9V O-serotype binding antibody levels (multiplex electrochemiluminescence-based immunoassay), 29 days post vaccination. Reactogenicity and safety were assessed. Results: Co-administration of ExPEC9V with HD influenza vaccine demonstrated non-inferiority (upper bound of 2-sided 95% confidence interval [CI] < 1.5 for HAI geometric mean ratio [Control/CoAd]) for all influenza strains. Non-inferiority for ExPEC9V O-serotype antibody levels was not demonstrated (upper bound 95% CI > 1.5). One of nine serotypes met the non-inferiority criterion; eight did not, with four narrowly failing to meet the non-inferiority criterion. ExPEC9V immunogenicity was similar regardless of urinary tract infection history. ExPEC9V was safe and well tolerated, with no serious adverse events related to ExPEC9V. Reactogenicity rate was higher with co-administration. Conclusions: Co-administration of ExPEC9V with HD influenza vaccine met non-inferiority criteria of humoral immune responses for influenza antigens, but not for ExPEC9V O-serotype antigens. ExPEC9V, administered alone or with HD influenza vaccine, was safe and well tolerated, with an acceptable reactogenicity profile.

## 1. Introduction

Extraintestinal pathogenic *Escherichia coli* (ExPEC) is an *E. coli* pathogroup that causes infections outside of the gastrointestinal tract [1]. These infections can lead to serious conditions such as urinary tract infections (UTIs), bloodstream infections, sepsis, and septic shock [1,2]. ExPEC, the leading cause of bacteremia in adults worldwide, is a major cause of community-onset sepsis, and subsequent hospitalization is associated with high morbidity and mortality [3,4].

Invasive *E. coli* disease (IED) is characterized as an acute illness indicative of systemic bacterial infection and is microbiologically confirmed by either the detection of *E. coli* in blood or other sterile body sites, or by the identification of *E. coli* in urine from a patient presenting with urosepsis when no alternative source of infection can be established [1,4,5]. Although IED affects all ages, older adults are at an increased risk of developing IED, including bacteremia and sepsis [1]. In a retrospective population-based study in the United States from 1998 to 2007, the incidence of *E. coli* bloodstream infections was shown to increase markedly in adults ≥ 60 years of age, with estimated incidence rates between 136 and 152 per 100,000 person-years in those aged ≥ 60 years, 100 per 100,000 person-years in those aged 60 to 79 years, and 300 per 100,000 person-years in those aged ≥ 80 years [6,7]. Notably, the most common primary source of infection was found to be urine (80.0%) [6,7].

Given the vulnerability of older adults to IED, ExPEC9V was developed as a 9-valent vaccine candidate for active immunization for the prevention of IED in adults aged ≥ 60 years. *E. coli* surface O-antigens serve as critical virulence factors that contribute to the pathogenicity of ExPEC [8,9]. ExPEC9V consists of the O-antigens that are among the most prevalent ExPEC serotypes associated with bloodstream infections [9] (O1A, O2, O4, O6A, O15, O16, O18A, O25B, and O75), each individually bioconjugated [4] to the carrier protein, a genetically detoxified form of exotoxin A derived from *Pseudomonas aeruginosa* (EPA). In a Phase 1/2a study, a 10-valent formulation of the vaccine was shown to be safe and immunogenic [10,11]. A Phase 3 efficacy study of ExPEC9V for the prevention of IED among older adults with a history of UTI in the prior 2 years was initiated in 2021 (E.mbrace; NCT04899336).

As older adults are recommended to receive multiple vaccines, including the seasonal influenza vaccine, the option to co-administer ExPEC9V with other vaccines would be advantageous. Co-administration of certain vaccines has been shown to be safe, effective, and acceptable to most people. No significant effect on immune response and higher reactogenicity rates were identified in a recent review [12]. However, because co-administration of vaccines has the potential to negatively impact immunogenicity, such studies are necessary as part of vaccine development [13]. Given the similar target population for ExPEC9V and the high-dose (HD) seasonal influenza vaccine, demonstrating safe and immunogenic co-administration could provide an efficient strategy to protect older adults against multiple infections.

To support potential co-administration, a Phase 3 study (E.ngage; NCT06134804) assessed the immunogenicity (humoral immune response to influenza strains and O-serotype antigens) and safety of ExPEC9V co-administered with a HD quadrivalent seasonal influenza vaccine (Co-administration [CoAd] group) compared with separate administrations 29 days apart (Control group) in adults aged ≥ 65 years. Because prior UTIs, particularly those attributable to *E. coli*, might induce higher pre-vaccination immune responses against *E. coli* antigens that could impact vaccine immunogenicity, this study further evaluated ExPEC9V immunogenicity in older adults with and without a history of UTI. This subgroup analysis aimed to determine whether the vaccine’s efficacy results, as evaluated in the separate Phase 3 efficacy study (E.mbrace), would also be applicable to a more general older adult population without a history of UTI.

## 2. Materials and Methods

### 2.1. Study Design, Treatments, and Participants

**Figure 1:**
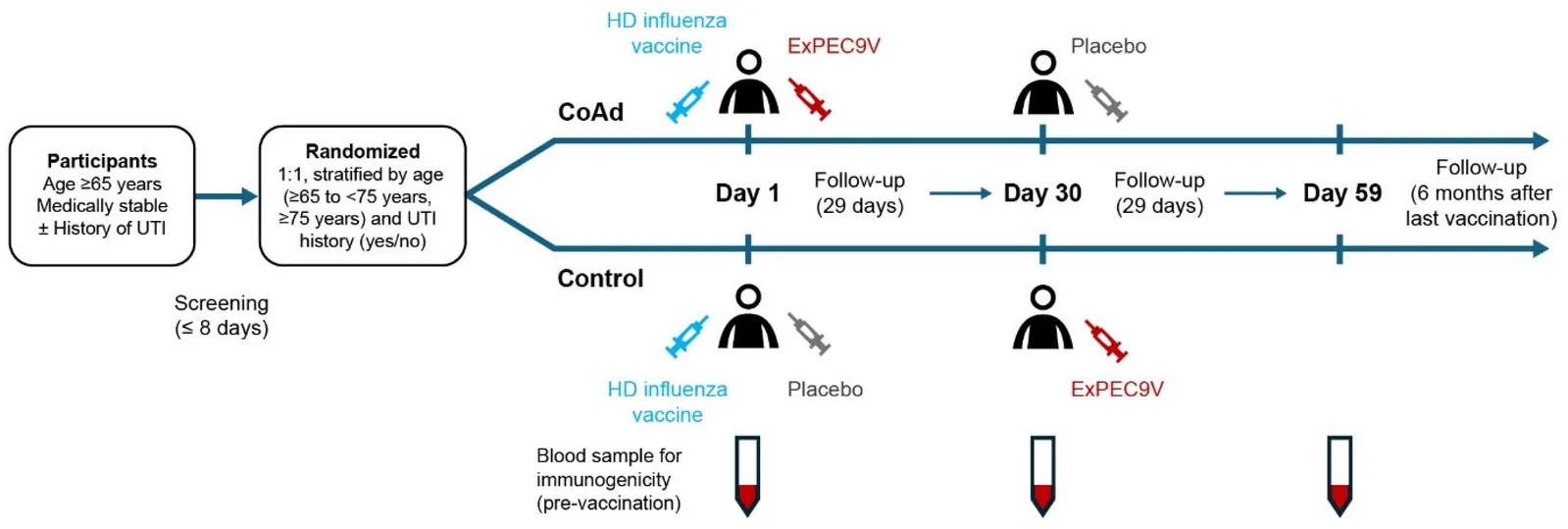
Study design. CoAd, co-administration; ExPEC9V, 9-valent extraintestinal pathogenic *Escherichia coli* vaccine; HD, high-dose; UTI, urinary tract infection.

This was a randomized, double-blind, placebo-controlled, parallel-group, multicenter, Phase 3 study in adult participants aged ≥ 65 years. The study was conducted between 24 October 2023 and 26 July 2024 (spanning influenza season in the Northern hemisphere) at 41 sites across Belgium, Canada, Poland, and the United States. A target of 932 participants was planned to be randomized in parallel in a 1:1 ratio to one of two vaccination groups (CoAd group, Control group). Randomization was stratified by age category (≥65 to <75 years, ≥75 years) and prior history of UTI (yes, no). All participants were centrally assigned/randomized to study vaccine using an interactive web response system. Participants in the CoAd group received blinded ExPEC9V and unblinded HD quadrivalent seasonal influenza vaccine in the contralateral arm on Day 1, administered in opposite arms, and blinded placebo on Day 30 (Figure 1).

Participants in the Control group received blinded placebo and unblinded HD quadrivalent seasonal influenza vaccine on Day 1, administered in contralateral arms, followed by blinded ExPEC9V on Day 30. Administration of ExPEC9V or placebo on Days 1 and 30 was performed in a blinded manner. An unblinded site pharmacist or other qualified individual prepared the labeled syringes (according to the randomization code) and provided them to the blinded vaccine administrator, who performed the injection. The left arm was used for administration of ExPEC9V or placebo; the contralateral arm received the unblinded influenza vaccine. Study vaccinations were given intramuscularly (IM). Study duration was ~7 months per participant. The study comprised a screening period within 8 days prior to randomization, study vaccinations on Days 1 and 30 with a 29-day follow-up period after each vaccination, and subsequent follow-up until 6 months after the last study vaccination.

This study was conducted in accordance with the ethical principles that have their origin in the Declaration of Helsinki and that are consistent with Good Clinical Practices and applicable regulatory requirements. Written informed consent was obtained from participants or their legally authorized delegate following disclosure of the study objectives, conditions for participation or withdrawal, and the potential risks and benefits associated with treatment. This study was registered with ClinicalTrials.gov (identifier: NCT06134804; date of registration: 13 November 2023) and the European Clinical Trial registration (EU CT number: 2023-504168-40-00; date of registration: 25 September 2023).

### 2.2. Inclusion and Exclusion Criteria

The study population consisted of adult participants aged ≥ 65 years who were in stable health based on the investigator’s clinical judgment, physical examination, medical history, and assessment of vital signs. Participants were specifically questioned regarding medical history of UTI, and individuals both with and without a history of UTI at any time prior to randomization were permitted to enter the study. See Supplementary Methods for the full list of inclusion and exclusion criteria.

### 2.3. Study Vaccine Administration and Dosing Details

The study vaccines were ExPEC9V (Johnson & Johnson; Bern, Switzerland) and Fluzone^®^ High-Dose Quadrivalent (Sanofi Pasteur Inc., Swiftwater, PA, USA; hereafter referred to as “HD influenza vaccine”). All study vaccinations were given IM as a single dose. ExPEC9V is an *E. coli* bioconjugate vaccine in phosphate-buffered solution containing nine O-antigen polysaccharides of ExPEC serotypes, O1A, O2, O4, O6A, O15, O16, O18A, O25B, and O75, individually bioconjugated to the carrier protein EPA, a genetically detoxified form of exotoxin A derived from *P. aeruginosa.* ExPEC9V was administered at a concentration of 176 µg polysaccharide/mL (0.5 mL). The HD influenza vaccine is indicated for active immunization against influenza A and B viruses in adults aged ≥ 65 years [14]. The HD influenza vaccine contains 240 µg of hemagglutinin—i.e., 60 µg of each of four influenza strains: two influenza A strains (A/Victoria/4897/2022 IVR-238 [H1N1] and A/Darwin/9/2021 SAN-010 [H3N2], hereafter referred to as A/Victoria [H1N1] and A/Darwin [H3N2], respectively) and two influenza B strains (B/Michigan/01/2021 wild type [B/Vic], and B/Phuket/3073/2013 wild type [B/Yam], hereafter referred to as B/Victoria and B/Yamagata, respectively). The influenza injection volume was 0.7 mL and placebo was normal saline (9 mg/mL sodium chloride [0.9% *w*/*v*]; 0.5 mL).

### 2.4. Immunogenicity

#### 2.4.1. Primary Objectives and Endpoints

The co-primary objectives were to demonstrate the non-inferiority of the humoral immune response to the four influenza vaccine strains after concomitant administration of ExPEC9V and HD influenza vaccine versus the administration of HD influenza vaccine administered alone, and the non-inferiority of the humoral immune response against the vaccine O-serotype antigens after concomitant administration of ExPEC9V and HD influenza vaccine versus the administration of ExPEC9V administered alone.

Primary endpoints were strain-specific influenza antibody titers assessed using a validated hemagglutination inhibition (HAI) assay against each of the four influenza vaccine strains, 29 days after the administration of HD influenza vaccine, and serotype-specific immunoglobulin G (IgG) binding antibody levels elicited by ExPEC9V, as determined by a validated multiplex electrochemiluminescence (ECL)-based immunoassay 29 days after administration of ExPEC9V (antibodies against the EPA carrier protein elicited by ExPEC9V were not included in the primary endpoint definition, but were also quantified by ECL assay). ECL testing was performed at Johnson & Johnson (Raritan, NJ, USA) according to previously published methods [10,11]. HAI testing was performed at Q Squared Solutions (Quest) LLC (Durham, NC, USA). Serum samples for determination of humoral immune responses were planned to be taken from all participants on Day 1 (before vaccination), Day 30, and Day 59.

#### 2.4.2. Secondary Objectives and Endpoints

Secondary objectives were to evaluate the immunogenicity of ExPEC9V between participants with a history of UTI and those without a history of UTI, and to further evaluate the immunogenicity of ExPEC9V when administered separately or concomitantly with HD influenza vaccine.

Secondary endpoints were serotype-specific total IgG levels and functional antibody titers elicited by ExPEC9V in participants with and without a history of UTI at enrollment. Serotype-specific total IgG antibody levels were determined by multiplex ECL-based immunoassay 29 days after administration of ExPEC9V. Functional opsonophagocytic antibody titers to vaccine O-serotype antigens were determined by a validated multiplex opsonophagocytic assay (MOPA) 29 days after administration of ExPEC9V. MOPA was performed at PPD Laboratories (Richmond, VA, USA).

### 2.5. Safety Assessments

The safety and reactogenicity of ExPEC9V when administered separately or concomitantly with HD influenza vaccine were assessed as a secondary endpoint. Safety assessments included solicited local (injection site) and systemic adverse events (AEs) recorded by participants in an electronic diary (eDiary) for 14 days after each vaccination, unsolicited AEs and special reporting situations for 29 days after each vaccination, medically attended adverse events (MAAEs) collected from administration of the first vaccine until 6 months after last vaccination, and serious adverse events (SAEs) collected over the same period. The severity of solicited signs and symptoms was graded by the participant in the eDiary using a scale based on an official toxicity grading guideline [15]. Unsolicited AEs with the onset date outside the timeframe defined above (>29 days after previous study vaccination), which were ongoing on the day of the subsequent vaccination, were recorded. MAAEs were defined as AEs with medically attended visits including hospital, emergency room, urgent care clinic, or other visits to or from medical personnel for any reason.

### 2.6. Statistical Analysis

A total of 419 evaluable participants per group were needed to have 99.91% power to demonstrate non-inferiority (see below for non-inferiority margin details) in HAI antibody titers for one influenza vaccine strain and to have 98.88% power to show non-inferiority in antibody levels against one O-serotype antigen, assuming no effect of co-administration on humoral immunogenicity assessments. With this sample size, the overall power to demonstrate non-inferiority in HAI antibody titers against each of the four influenza vaccine strains at 29 days after the administration of HD influenza vaccine, as well as non-inferiority in antibody levels against each of the nine O-serotype antigens at 29 days after the administration of ExPEC9V, was ≥90%. To account for an anticipated 10% exclusion rate from the per-protocol set, drop-outs and missing samples, ~466 participants per group were planned to be enrolled, resulting in a total target enrollment of ~932 participants.

The full analysis set (FAS) included all participants who received at least one study vaccination, regardless of the occurrence of protocol deviations and vaccine type (HD influenza vaccine, ExPEC9V, or placebo). All safety demographic data analyses were based on the FAS. The Per-Protocol Influenza Immunogenicity (PPII) Set included all randomized participants who received the first study vaccination and for whom immunogenicity data were available for at least one influenza strain. The Per-Protocol ExPEC9V Immunogenicity (PPEI) Set included all randomized participants who received ExPEC9V, either concomitantly with HD influenza vaccine (CoAd group) or alone (Control group) and for whom O-antigen immunogenicity data were available.

The primary immunogenicity objectives were assessed by calculating the 2-sided 95% confidence intervals (CIs) for the differences between the Control and CoAd groups in (1) log10-transformed HAI antibody titers against each of the four influenza vaccine strains at 29 days after the administration of the HD influenza vaccine, and (2) log10-transformed O-serotype antibody levels against each of the nine O-serotypes at 29 days after the administration of ExPEC9V.

For both HAI antibody titers and O-serotype antibody levels, an analysis of variance (ANOVA) model was fitted (using SAS proc mixed, version 9.4) with influenza strain titers and O-serotype levels as dependent variables, and treatment group (Control or CoAd), age category, and history of UTI (as collected) as fixed effects. Based on these ANOVA models, 2-sided 95% CIs around the between-group difference (Control group minus CoAd group) were calculated and back-transformed to derive CIs around a geometric mean titer (GMT) ratio (Control group/CoAd group) and compared to the non-inferiority margin of 1.5. Least squares (LS) means and corresponding CI of the log-transformed HAI antibody titers, and ExPEC9V O-serotype antibody levels, were back-transformed (by exponentiation) to GMTs. Titer/levels below the lower limit of quantification (LLOQ) were replaced by one-half the LLOQ. For calculation of geometric mean fold increase (GMFI), titer values/antibody levels below the LLOQ were replaced with the LLOQ. Titer/levels above the upper limit of quantification (ULOQ) were replaced by the ULOQ. Missing immunogenicity data were not imputed.

Non-inferiority of co-administration versus separate administration could only be concluded for both vaccines (HD influenza vaccine and ExPEC9V) if the upper bound of the 2-sided 95% CI was <1.5 for the GMT ratio (Control group/CoAd group) of the HAI antibody titers for each of the four influenza vaccine strains and of the O-serotype antibody levels for each of the nine O-serotype antigens as measured by ECL. Non-inferiority could not be concluded if any upper confidence limit for the GMT ratio exceeded 1.5.

Non-inferiority of co-administration versus separate administration of ExPEC9V and HD influenza vaccine was also assessed based on opsonophagocytic activity (MOPA; secondary objective). Similar to the primary endpoints, a non-inferiority margin of 1.5 was applied for the GMT ratio (Control group/CoAd group) of MOPA titers.

For ExPEC9V O-serotype antibodies as measured by the multiplex ECL-based immunoassay and MOPA, and EPA antibodies as measured by the multiplex ECL-based immunoassay only, immunogenicity included GMTs at all timepoints, the GMFI from baseline to 29 days, and the proportion of participants with a ≥2-fold increase in serum antibody levels from baseline to 29 days after vaccination. These measures were also assessed by study group stratified by history of UTI.

AEs were coded in accordance with Medical Dictionary for Regulatory Activities version 26.1. The number and percentage of participants with at least one specific AE (solicited/unsolicited) were summarized by class (local or systemic) and preferred term (PT). Unsolicited AEs, SAEs, and MAAEs were summarized by system organ class (SOC) and PT. No formal statistical testing of the safety endpoints was performed; all data were summarized descriptively.

## 3. Results

### 3.1. Participant Disposition, Demographic, and Baseline Characteristics

**Figure 2:**
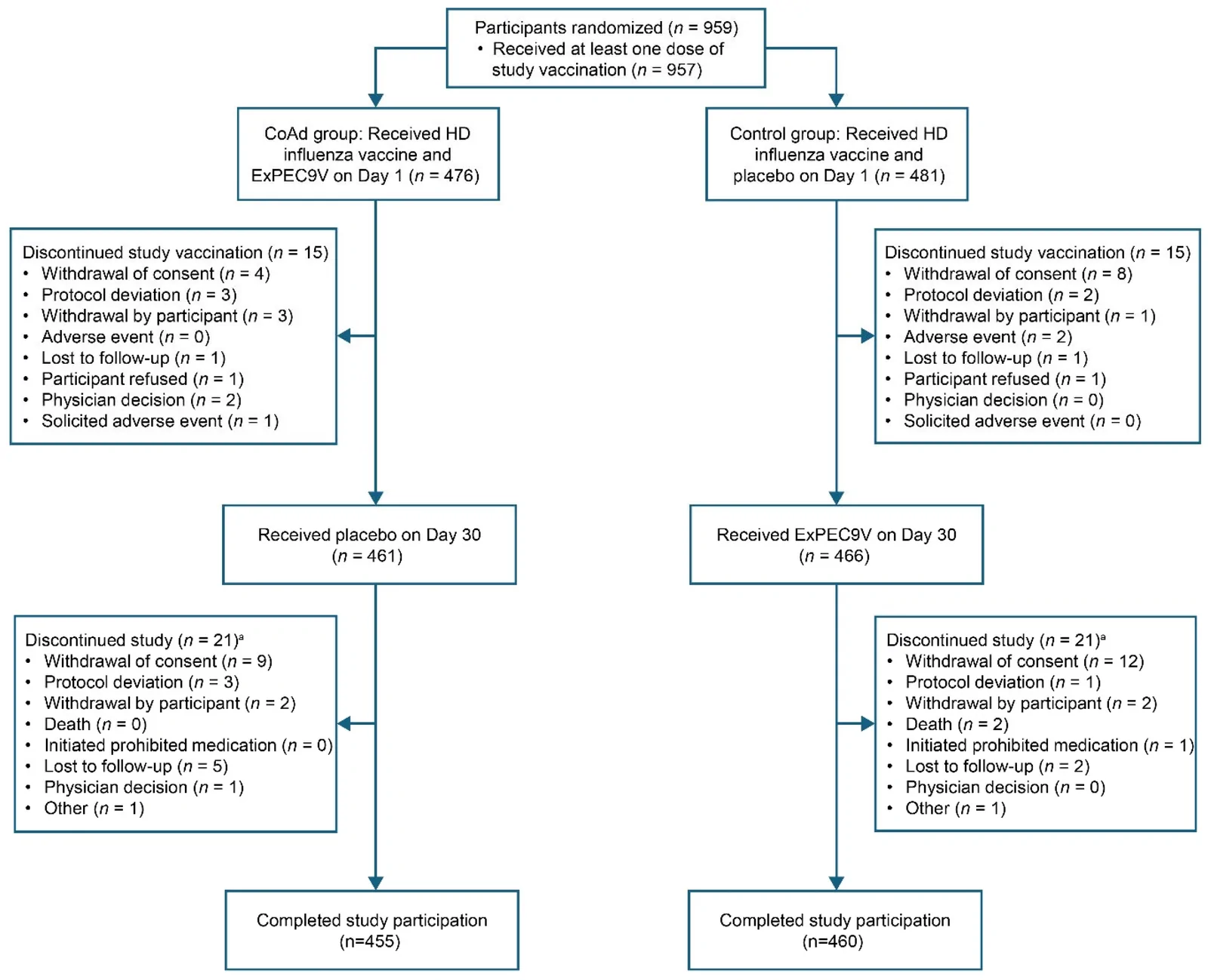
Participant flow diagram. In the CoAd group, 8 participants discontinued the study but completed vaccination, and 2 discontinued vaccination but completed the study. In the Control group, 8 participants discontinued the study but completed vaccination, and 2 discontinued vaccinations but completed the study. ^a^ Includes participants who discontinued study vaccination. CoAd: ExPEC9V + HD influenza vaccine (Day 1), placebo (Day 30). Control: placebo + HD influenza vaccine (Day 1), ExPEC9V (Day 30).

A total of 959 participants were randomized, of whom 957 received at least one dose of study vaccine and were included in the FAS (Figure 2); there were 476 participants in the CoAd group and 481 in the Control group. A total of 461 (96.8%) participants in the CoAd group and 466 (96.9%) in the Control group completed the study vaccination (i.e., received two doses of study vaccine per protocol). A total of 915 (95.6%) participants completed the study; the most common reason for study discontinuation was withdrawal of consent (21 [2.2%] participants). Of the 957 participants who received the first study vaccination, 30 (3.1%) did not receive their second study vaccination (15 [3.2%] and 15 [3.1%] participants in the CoAd and Control groups, respectively). The most common reason for discontinuation of study vaccination was withdrawal of consent (4 [0.8%] and 8 [1.7%], participants in the CoAd and Control groups, respectively); 1 (0.2%) participant in the CoAd group discontinued study vaccination due to a solicited AE, and 2 (0.4%) in the Control group discontinued study vaccination due to AEs. At 29 days after HD influenza vaccination, samples for immunogenicity analysis were available for 937 participants (468 in the CoAd group and 469 in the Control group) in the PPII analysis set. At 29 days after ExPEC9V vaccination, samples for immunogenicity analysis were available for 932 participants (468 in the CoAd and 464 in the Control group) in the PPEI analysis set.

Demographic and baseline characteristics were generally well balanced between the CoAd and Control groups (Table 1). The median (range) age was 71 (65–93) years, with 75.9% of participants aged 65–74 years, and 24.1% aged ≥ 75 years. A total of 55.3% of participants were male and 90.7% were White. Overall, 24.8% of participants reported a history of UTI at baseline.

### 3.2. Immunogenicity

#### 3.2.1. Immune Responses to HD Influenza Vaccine

**Figure 3:**
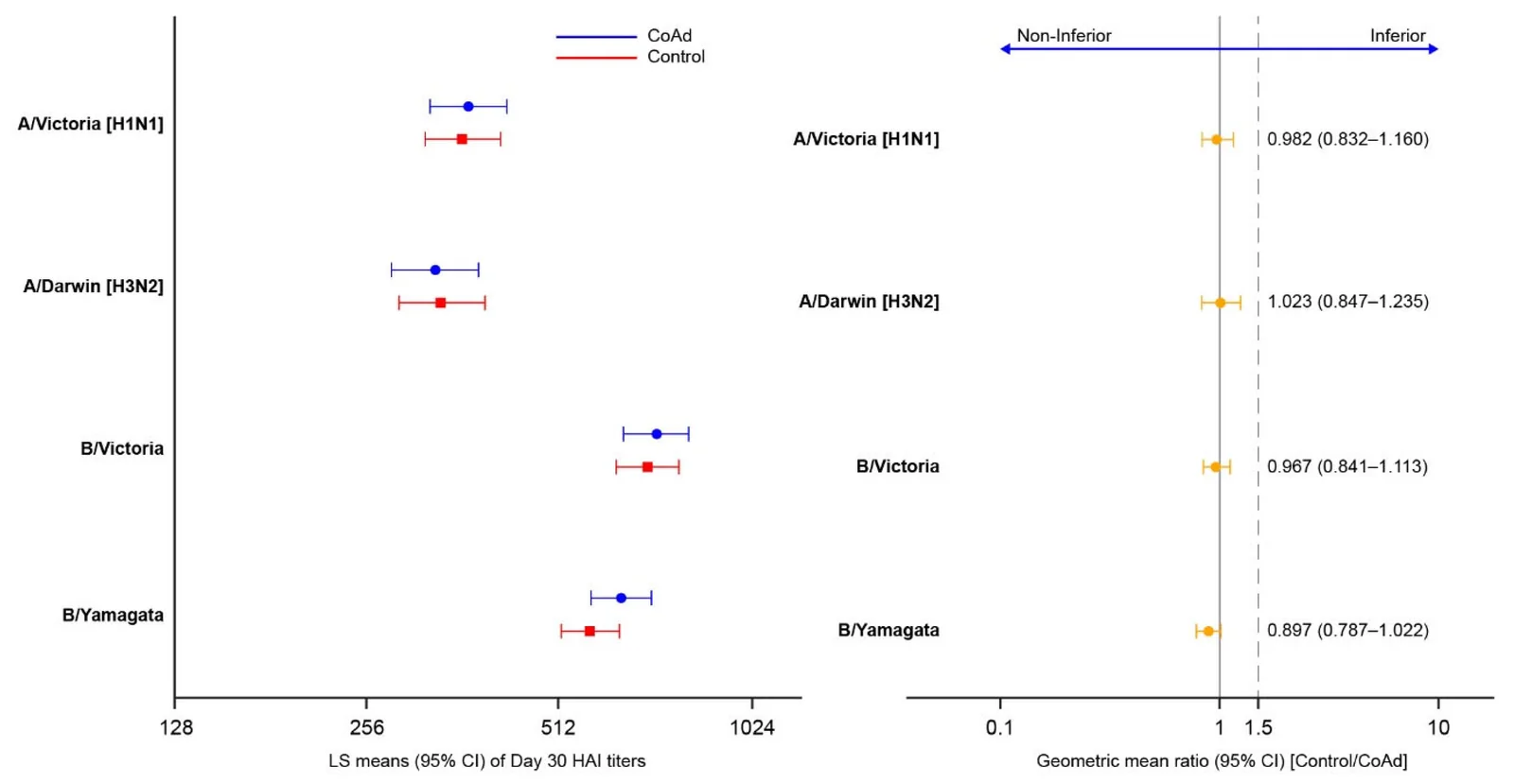
HAI antibody response. LS means, and corresponding CIs of the log-transformed HAI titers, were back-transformed (by exponentiation) to a GMT. CoAd: ExPEC9V + HD influenza vaccine (Day 1), placebo (Day 30). Control: placebo + HD influenza vaccine (Day 1), ExPEC9V (Day 30). A/Victoria [H1N1], A/Victoria/4897/2022 IVR-238; A/Darwin [H3N2], A/Darwin/9/2021; B/Victoria, B/Michigan/1/2021; B/Yamagata, B/Phuket/3073/2013; CI, confidence interval; HAI, hemagglutination inhibition assay; LS, least squares.

The immune response to the HD influenza vaccine met the primary immunogenicity objective for all four influenza vaccine strains. Non-inferiority of concomitant administration of the HD influenza vaccine and ExPEC9V versus administration of the HD influenza vaccine alone was demonstrated 29 days after administration of the HD influenza vaccine, with the upper bounds of the 95% CIs for the GMT ratios (Control group/CoAd group) being below the predefined non-inferiority margin of 1.5 ranging from 0.897 (95% CI 0.787–1.022) for B/Yamagata to 1.023 (95% CI 0.847–1.235) for A/Darwin (H3N2) (Figure 3 and Table S1).

#### 3.2.2. Immune Responses to ExPEC9V: Binding Antibodies

**Figure 4:**
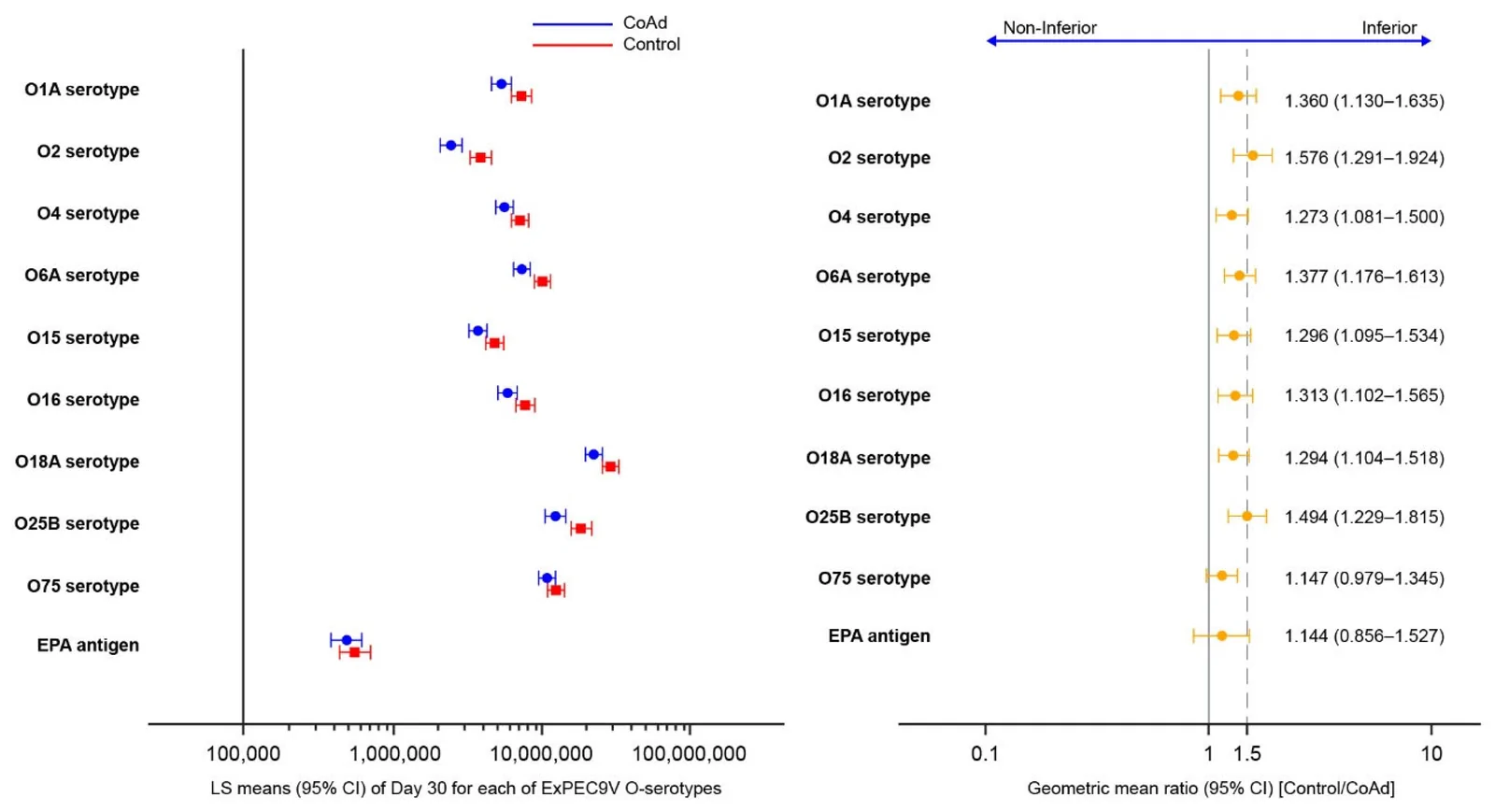
ExPEC9V O-serotype multiplex ECL-based immunoassay antibody response. LS means, and corresponding CIs of the log-transformed ExPEC9V O-serotype titers, were back-transformed (by exponentiation) to a GMT. Non-inferiority assessment of EPA-induced antibodies was not included in the primary endpoint definition. CoAd: ExPEC9V + HD influenza vaccine (Day 1), placebo (Day 30). Control: placebo + HD influenza vaccine (Day 1), ExPEC9V (Day 30). ECL, electrochemiluminescence.

The immune response to ExPEC9V met the primary immunogenicity objective for one of the nine vaccine O-serotypes. For serotype O75, non-inferiority of concomitant administration of ExPEC9V and the HD influenza vaccine versus administration of ExPEC9V alone was demonstrated 29 days after administration of ExPEC9V, with the upper bound of the 95% CI for the GMT ratio (Control group/CoAd group) being below the predefined non-inferiority margin of 1.5 (1.147 [95% CI 0.979–1.345]) as measured by ECL (Figure 4). For four additional serotypes (O4, O15, O16, and O18A), the upper bound of the 95% CI for the GMT ratio slightly exceeded the non-inferiority margin (1.500–1.565), and for the remaining four serotypes (O1A, O2, O6A, and O25B), non-inferiority was not met (upper bounds 1.613–1.924) (Figure 4). Although not part of the primary objective, the study also narrowly failed to demonstrate non-inferiority for the response to the carrier protein EPA, with the upper bound of the 95% CI for the GMT ratio (Control group/CoAd group) slightly higher than the non-inferiority margin of 1.5 (i.e., 1.527).

**Figure 5:**
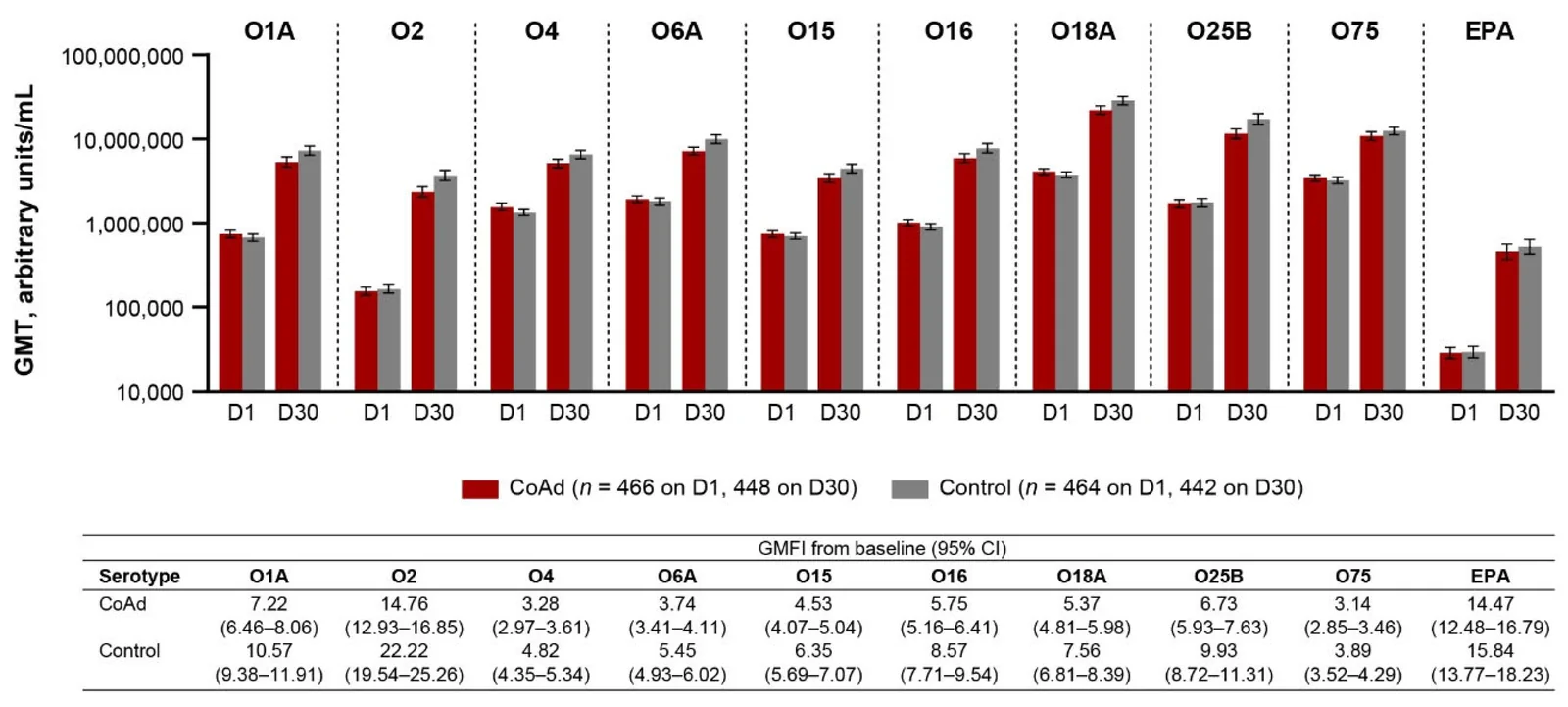
ExPEC9V O-serotype multiplex ECL-based immunoassay antibody responses on Day 1 and Day 30 (PPEI) and GMFI (95% CI) from baseline in ExPEC9V O-serotype total (IgG) antibody levels. Error bars represent the 95% CIs for the GMT. 95% CIs for the GMT and GMFI are based on the t-distribution. CoAd: ExPEC9V + HD influenza vaccine (Day 1), placebo (Day 30). Control: placebo + HD influenza vaccine (Day 1), ExPEC9V (Day 30). D, Day; EPA, ExoProtein A; GMFI, geometric mean fold increase; GMT, geometric mean titer; PPEI, Per-Protocol ExPEC9V Immunogenicity Set.

Baseline antibody levels (GMTs), as measured by ECL assay, for all nine O-serotypes and EPA were comparable between the CoAd and Control groups, with both groups exhibiting an increase in antibody levels 29 days post vaccination with ExPEC9V (Figure 5). The observed antibody levels and GMFI from baseline to 29 days post vaccination with ExPEC9V were generally lower in the CoAd group compared to the Control group across all nine O-serotypes and EPA (Figure 5). In the CoAd group, GMFI ranged from 3.28 for serotype O4 to 14.76 for serotype O2, whereas in the Control group they ranged from 3.89 for serotype O75 to 22.22 for serotype O2. The 95% CIs of the GMFI overlapped between the two vaccination groups only for EPA. The proportions of participants with a ≥2-fold increase in antibody levels, 29 days after ExPEC vaccination, were similar between groups for serotypes O1A, O2, O18A, O75, and EPA (Table S2); whereas lower proportions were observed in the CoAd group compared to the Control group for serotypes O4, O6A, O15, O16, and O25B.

#### 3.2.3. Immune Responses to ExPEC9V: Functional Antibodies

In terms of the MOPA results, the response to ExPEC9V met the non-inferiority immunogenicity objective for two of the nine vaccine O-serotypes. For serotypes O4 and O6A, non-inferiority of concomitant administration of ExPEC9V with the HD influenza vaccine versus administration of ExPEC9V alone was demonstrated 29 days after administration of ExPEC9V, with the upper bounds of the 95% CIs for the GMT ratios (Control group/CoAd group) being below the predefined non-inferiority margin of 1.5 (Table S3). The antibody GMT ratios by MOPA, 29 days after administration of ExPEC9V, were 1.221 (95% CI 1.028–1.450) for serotype O4 and 1.130 (95% CI 0.935–1.366) for serotype O6A. For the remaining serotypes (O1A, O2, O15, O16, O18A, O25B, and O75), non-inferiority was not demonstrated, with the upper bounds of the 95% CI for the GMT ratios (Control group/CoAd group) ranging from 1.624 to 2.139. Functional antibody levels (GMTs) for most O-serotypes were slightly higher at baseline and slightly lower 29 days post vaccination with ExPEC9V in the CoAd group compared with the Control group (Figure S1). The GMFI ranged from 2.05 for serotype O25B to 9.59 for serotype O16 in the CoAd group, and from 2.77 for serotype O25B to 17.02 for serotype O16 in the Control group. The 95% CIs of the GMFI did not overlap between the two vaccination groups for any of the O-serotypes.

#### 3.2.4. History of UTI and Immune Responses to ExPEC9V

To evaluate whether a history of UTI affected ExPEC9V-induced immune responses, immunogenicity was compared between participants with and without a history of UTI. As non-inferiority of concomitant administration was not demonstrated for ExPEC9V, the analysis focused on the Control group. In the Control group, ExPEC9V immunogenicity was similar irrespective of UTI history. Mean serotype- and EPA-specific antibody levels at baseline and 29 days after ExPEC9V vaccination were similar in participants with and without a history of UTI (Figure S2). Likewise, the proportion of participants with a ≥2-fold increase in antibody levels was similar across both groups (Table S4).

Consistent findings were observed in the MOPA analysis. For all nine serotypes, the 95% CIs overlapped between participants with and without history of UTI, both at baseline and 29 days post vaccination with ExPEC9V (Figure S3). Additionally, the GMFIs 29 days post vaccination with ExPEC9V were comparable, regardless of participant’s history of UTI (Figure S3).

### 3.3. Safety

Safety analyses were based on the 957 participants included in the FAS (476 and 481 in the CoAd and Control groups, respectively) (Table 2 and Tables S5–S8).

#### 3.3.1. Solicited AEs

Irrespective of injection site, solicited local AEs were reported in a higher percentage of participants in the CoAd group after co-administration of ExPEC9V and HD influenza vaccine (64.9%) than in the Control group after administration of ExPEC9V alone (35.2%) or HD influenza vaccine and placebo (54.3%) (Table 2). However, when analyzed by injection site, solicited local AEs were reported in a similar percentage of participants in the CoAd and Control groups (ExPEC9V injection site: CoAd group, 42.6%; Control group, 35.2%; HD influenza vaccine injection site: CoAd group, 45.8%; Control group, 48.0%; Table S5).

Following ExPEC9V administration, the median time to onset of each solicited local AE at the ExPEC9V injection site ranged from 6 to 8 days in the CoAd group and from 7 to 8 days in the Control group (Table S6), with median durations of 3 and 2 days, respectively.

Late solicited local AEs (i.e., onset after Day 5 post vaccination) at the ExPEC9V injection site were reported in 113 (23.7%) participants in the CoAd group and 122 (26.2%) in the Control group (Table S6). The most frequent late solicited local AE was pain, reported in 112 (23.5%) and 120 (25.8%) participants in the CoAd and Control groups, respectively. The majority of late-onset solicited local AEs were grade 1 or 2 in severity and had a median duration of 3 days (CoAd group) and 2 days (Control group).

Solicited systemic AEs were reported more frequently in the CoAd group (53.8%) than in the Control group after administration of ExPEC9V alone (36.9%) or after HD influenza vaccine plus placebo (44.9%) (Table 2 and Table S7). The most frequently reported (>15% after any study vaccination) solicited systemic AEs were fatigue, myalgia, and headache. Most were grade 1 or 2 in severity, and no grade 4 events were reported (Table S7).

#### 3.3.2. Unsolicited AEs

Unsolicited AEs occurred in a similar percentage of participants in the CoAd group (15.3%), in the Control group after ExPEC9V (15.0%), and in the Control group after HD influenza vaccine plus placebo (17.7%) (Table 2). The most frequently reported unsolicited AEs were nasopharyngitis and COVID-19; no other unsolicited AE by PT was reported in ≥2% of participants after any study vaccination (Table S8).

#### 3.3.3. MAAEs

Overall, MAAEs were reported in 82 (17.2%) participants in the CoAd group and 85 (17.7%) in the Control group. The most frequently reported MAAEs (>1% of participants in either the CoAd or Control group) were UTI (1.7% and 1.9%, respectively), upper respiratory tract infection (0.8% and 1.2%, respectively), hypertension (1.3% and 0.6%, respectively), sinusitis (0 and 1.5%, respectively), and cataract (0.2% and 1.2%, respectively). No pattern was observed in SOC or PTs. MAAEs considered by the investigator to be related to study vaccine were reported in 4 (0.8%) participants in the Control group. Of these, three events (vaccination site inflammation, transient ischemic attack, and rash in 1 participant each) occurred after the first study vaccination (before ExPEC9V vaccination), and one related event (arthralgia) occurred after ExPEC9V vaccination. Except for the transient ischemic attack (discussed in Section 3.3.4), the related MAAEs were non-serious and grade 1 or 2 in severity. No related MAAEs were reported in the CoAd group.

#### 3.3.4. SAEs and Deaths

Overall, SAEs were reported in 13 (2.7%) participants in the CoAd group and 11 (2.3%) in the Control group. No pattern was observed in the SOC or PTs of reported SAEs. One SAE—transient ischemic attack—occurred in the Control group 8 days after administration of HD influenza vaccine alone (i.e., prior to ExPEC9V exposure) and was considered by the investigator to be related to study vaccine. No SAEs related to ExPEC9V were reported.

Two deaths were reported during the study, both considered by the investigator to be unrelated to study vaccine. One participant in the Control group with a history of recurrent UTI and urinary incontinence received study vaccinations on Days 1 and 30. A grade 2 urinary retention AE, deemed unrelated to the vaccine, occurred on Day 37 and resolved after 13 days. A grade 4 UTI SAE was reported on Day 53, leading to death 57 days later. One participant in the Control group died from an unknown cause. The participant received study vaccination on Days 1 and 30. Death was reported on Day 64, with no other unsolicited AEs or information on the cause.

#### 3.3.5. AEs Leading to Discontinuation of Study Vaccination

Solicited local AEs leading to discontinuation of study vaccine were reported in one participant in the CoAd group after the first study vaccination. Grade 2 vaccination-site erythema and swelling were reported at the ExPEC9V injection site on Day 6 and, as local AEs, were considered by definition to be related to study vaccine. Both events were resolved after 4 days.

Unsolicited AEs leading to discontinuation of study vaccine were reported in 2 participants in the Control group after the first study vaccination with HD influenza vaccine and placebo—grade 3 pneumonitis (occurring 13 days post vaccination with HD influenza vaccine) and grade 2 deep vein thrombosis (occurring 24 days post vaccination with HD influenza vaccine) reported in 1 participant each. Both events were resolved and were considered by the investigator to be unrelated to study vaccine.

## 4. Discussion

This study addressed a key question in the clinical development of ExPEC9V: whether the vaccine candidate can be co-administered with a HD seasonal influenza vaccine in older adults without compromising immunogenicity or safety. Older adults represent the principal target population for ExPEC vaccination because of their increased risk of IED, including bacteremia and sepsis [6,7]. The overall case fatality rate across all age groups is ~12% [3].

Previously published research evaluating vaccines targeting the ExPEC O-antigen—a component of surface lipopolysaccharide—has shown these vaccines to be both safe and immunogenic in humans when administered alone [8,10,16,17]. Because seasonal influenza vaccination is routinely recommended for older adults, to improve vaccination program convenience and efficiency it is important to assess whether ExPEC9V can be co-administered with influenza vaccines without immune interference or with an acceptable reactogenicity profile [12]. This Phase 3 randomized controlled trial is, to our knowledge, the first to evaluate the immunogenicity and safety of an ExPEC vaccine co-administered with an HD quadrivalent seasonal influenza vaccine compared with separate administration, to support the potential concomitant administration of ExPEC9V and influenza vaccination in the older adult target population.

In this study, non-inferiority of the immune response to influenza antigens was demonstrated for all four tested influenza strains, indicating that ExPEC9V co-administration does not impair influenza vaccine immunogenicity. In contrast, non-inferiority for ExPEC9V O-serotype antigens was not demonstrated 29 days after co-administration of ExPEC9V with HD influenza vaccine; one of nine O-serotypes met non-inferiority criteria and eight serotypes did not, with four serotypes narrowly missing the predefined margin. Mean ExPEC9V-specific IgG levels were lower when ExPEC9V was co-administered with the HD influenza vaccine than when ExPEC9V was given alone. Functional antibody results (MOPA) were generally concordant with those of the ECL IgG data. Functional antibody titers were lower in the CoAd group than in the Control group, and for the majority of serotypes, non-inferiority by MOPA for ExPEC9V O-serotype antigens 29 days after ExPEC9V vaccination was not met. Although co-administration reduced ExPEC9V antibody responses as measured by both assays, the IgG and functional antibody levels required for protection against IED remain unknown, and the clinical significance of this reduction is therefore uncertain. The modestly lower responses may reflect immune interference, as occasionally observed with co-administration of other conjugate or polysaccharide-based vaccines [18,19]. While within this study ExPEC9V co-administration with HD influenza vaccine was shown to be safe, it is unknown whether further increasing the dose of ExPEC9V would lead to heightened immune response to ExPEC9V O-serotypes and allow for co-administration with the influenza vaccine.

ExPEC9V-induced immunogenicity was similar irrespective of UTI history. Baseline and post vaccination serotype-specific IgG levels were comparable between participants in the Control group with and without a history of UTI, aligning with findings from a previous study assessing the safety and immunogenicity of ExPEC10V, and a 10-valent *E. coli* bioconjugate vaccine, in healthy older adults with a history of UTI [11]. In a small cohort of participants, a robust immune response was observed following ExPEC10V vaccination [11]. These results suggest that a history of UTI, which in the majority of cases is caused by *E. coli*, does not appear to influence vaccine-induced antibody responses and that immunogenicity findings are likely generalizable to a broader older adult population. Potential mechanisms for interference include innate immune competition and antigenic load effects in an immunosenescent population. Importantly, the ExPEC10V data in older adults, including cohorts with UTI history, showed robust responses when the ExPEC vaccine was administered alone; the current results, therefore, likely reflect a co-administration effect rather than an intrinsic lack of immunogenicity.

With respect to safety, this Phase 3 study demonstrated that co-administration of both vaccines was generally safe and well tolerated in older adults. ExPEC9V had an acceptable safety profile when administered alone or concomitantly with HD influenza vaccine. Late (>5 days post vaccination) reactogenicity was observed, consistent with earlier ExPEC4V and ExPEC10V clinical trials and also with other conjugate vaccines [10,16,17]. The frequency and severity of these events were similar between the CoAd and Control groups. Despite slightly higher overall reactogenicity rates with co-administration, the reactogenicity profile of ExPEC9V remained acceptable. No ExPEC9V-related SAEs were reported.

The strengths of this study include its randomized, double-blind, placebo-controlled, parallel-group, multicenter design and inclusion of adults aged ≥ 65 years. The study achieved a high retention rate, minimizing responder bias, and the study populations were well balanced between the Control and CoAd groups, with appropriate stratification by age (≥65 to <75 years, ≥75 years).

Some study design aspects may have influenced the results. Sample size calculations were based on ECL and HAI endpoints and assumed that co-administration of ExPEC9V and HD influenza vaccine would not reduce antibody levels. Co-administration of ExPEC9V with HD influenza vaccine preserved influenza HAI responses but attenuated ExPEC9V serotype-specific IgG and functional responses relative to separate administration, and the study did not meet non-inferiority for most ExPEC antigens. These findings are consistent with reports of modest antigen-specific attenuation seen in adult studies of co-administration of multivalent pneumococcal conjugate vaccines (PCVs) [18,20,21]. In those studies of co-administration of 13-valent [21] and 20-valent [20] pneumococcal conjugate vaccines (PCV13 and PCV20), serotype-specific antibody responses were slightly lower when co-administered with influenza vaccines in older adults; yet, non-inferiority was met for most serotypes using the wider non-inferiority margin of 2.0. In the current study, the more stringent non-inferiority margin of 1.5-fold was chosen according to the World Health Organization (WHO) recommendation. A descriptive sensitivity using a margin of 2.0 suggests several borderline ExPEC9V O-serotypes would have met non-inferiority; however, conclusions remain anchored to the prespecified 1.5 margin. The more conservative 1.5-fold margin and modest sample size in this study may therefore have contributed to the failure to meet the ExPEC9V non-inferiority criterion.

Additional limitations relate to the functional antibody (MOPA) analyses. In February 2025, following an interim efficacy analysis on the Phase 3 ExPEC9V E.mbrace trial, the sponsor terminated the clinical program due to insufficient efficacy for prevention of IED. Consequently, MOPA testing for serotypes O15, O18A, and O75 was not completed. Baseline titer differences between groups further complicated the interpretation of results. However, a sensitivity analysis adjusting for baseline titers confirmed that non-inferiority was not demonstrated for any O-serotype. These study limitations may have impacted the immunogenicity results, but are unlikely to have influenced the overall immunogenicity conclusions with regard to ExPEC9V.

## 5. Conclusions

Co-administration of ExPEC9V and HD influenza vaccine in older adults was safe and well tolerated and did not affect influenza vaccine immunogenicity. However, ExPEC9V antibody responses, as measured by both ECL and MOPA, were reduced and non-inferiority was not demonstrated for most O-serotypes. Although these findings support the safety of co-administration, the immunogenicity data suggest that HD quadrivalent seasonal influenza vaccine and ExPEC9V vaccine should not be administered concomitantly. The ExPEC9V clinical program has been discontinued, but these results remain informative for future development of multivalent *E. coli* conjugate vaccines targeting invasive disease. They also highlight the importance of selecting an appropriate non-inferiority margin and adequate sample size when designing co-administration trials.

## Figures and Tables

**Table 1:** Demographic and baseline characteristics (FAS).

Characteristic	CoAd *N* = 476	Control *N* = 481	Total *N* = 957
Age, years			
Mean (SD)	71.5 (5.08)	71.8 (4.89)	71.6 (4.99)
Median (range)	70 (65–93)	71 (65–90)	71 (65–93)
65–74 years	362 (76.1)	364 (75.7)	726 (75.9)
≥75 years	114 (23.9)	117 (24.3)	231 (24.1)
Sex			
Male	265 (55.7)	264 (54.9)	529 (55.3)
Female	211 (44.3)	217 (45.1)	428 (44.7)
Race			
White	438 (92.0)	430 (89.4)	868 (90.7)
Other	38 (8.0)	51 (10.6)	89 (9.3)
Country			
Belgium	68 (14.3)	73 (15.2)	141 (14.7)
Canada	36 (7.6)	34 (7.1)	70 (7.3)
Poland	125 (26.3)	126 (26.2)	251 (26.2)
USA	247 (51.9)	248 (51.6)	495 (51.7)
History of UTI (baseline)			
Yes	116 (24.4)	121 (25.2)	237 (24.8)
Male	23 (4.8)	17 (3.5)	40 (4.2)
Female	93 (19.5)	104 (21.6)	197 (20.6)

Data are *n* (%), except where noted. CoAd: ExPEC9V + HD influenza vaccine (Day 1), placebo (Day 30). Control: placebo + HD influenza vaccine (Day 1), ExPEC9V (Day 30). FAS, full analysis set; SD, standard deviation; USA, United States of America.

**Table 2:** Solicited and unsolicited AEs (FAS).

AE Type, *n* (%)	Post Vaccination 1		Post Vaccination 2	
	ExPEC9V + HD Influenza Vaccine (CoAd) *N* = 476	Placebo + HD Influenza Vaccine (Control) *N* = 481	Placebo (CoAd) *N* = 461	ExPEC9V (Control) *N* = 466
Solicited AE	358 (75.2)	319 (66.3)	137 (29.7)	219 (47.0)
Worst grade 3	22 (4.6)	14 (2.9)	1 (0.2)	18 (3.9)
Solicited local AE	309 (64.9)	261 (54.3)	55 (11.9)	164 (35.2)
HD influenza vaccine injection site	218 (45.8)	231 (48.0)	NA	NA
ExPEC9V/placebo injection site	203 (42.6)	94 (19.5)	55 (11.9)	164 (35.2)
Solicited local AE of worst grade 3	17 (3.6)	0	1 (0.2)	10 (2.1)
HD influenza vaccine injection site	3 (0.6)	0	NA	NA
ExPEC9V/placebo injection site	15 (3.2)	0	1 (0.2)	10 (2.1)
Solicited systemic AE	256 (53.8)	216 (44.9)	120 (26.0)	172 (36.9)
Worst grade 3	8 (1.7)	14 (2.9)	1 (0.2)	10 (2.1)
Related to study vaccine	233 (48.9)	192 (39.9)	93 (20.2)	144 (30.9)
Unsolicited AE	73 (15.3)	85 (17.7)	62 (13.4)	70 (15.0)
Worst grade 1	37 (7.8)	41 (8.5)	36 (7.8)	41 (8.8)
Worst grade 2	32 (6.7)	36 (7.5)	23 (5.0)	25 (5.4)
Worst grade 3	4 (0.8)	6 (1.2)	3 (0.7)	2 (0.4)
Worst grade 4	0	0	0	2 (0.4)
Related to study vaccine	12 (2.5)	13 (2.7)	4 (0.9)	8 (1.7)
Grade 1	11 (2.3)	10 (2.1)	2 (0.4)	7 (1.5)
Grade 2	2 (0.4)	2 (0.4)	1 (0.2)	1 (0.2)
Grade 3	0	1 (0.2)	1 (0.2)	0

Participants are counted only once for any given event, regardless of the number of times they actually experienced the event. CoAd: ExPEC9V + HD influenza vaccine (Day 1), placebo (Day 30). Control: placebo + HD influenza vaccine (Day 1), ExPEC9V (Day 30). AE, adverse event; NA, not applicable.

## Data Availability

The data sharing policy of Johnson & Johnson is available at https://www.jnj.com/innovativemedicine/our-innovation/clinical-trials/transparency (accessed on 26 January 2026). As noted on this site, requests for access to the study data can be submitted through Yale Open Data Access (YODA) Project site at http://yoda.yale.edu (accessed on 26 January 2026).

## Supplementary Materials

The following supporting information can be downloaded at: https://www.mdpi.com/article/10.3390/vaccines14020146/s1, Supplementary Methods; Table S1: HAI antibody response to influenza strains: Comparison between groups (Control/CoAd) using age and history of UTI as collected (PPII); Table S2: Number and percentage of participants with a ≥2-fold increase in total (IgG) antibody levels 29 days post vaccination with ExPEC9V (PPEI); Table S3: O-Serotype MOPA antibody response (PPEI); Table S4: Number and percentage of participants with a ≥2-fold increase in total (IgG) antibody levels 29 days post vaccination with ExPEC9V by ECL by history of UTI (PPEI); Table S5: Solicited local adverse events by derived term and worst severity (FAS); Table S6: Duration and timing of solicited local adverse events by derived term (FAS); Table S7: Solicited systemic adverse events by derived term and worst severity grade (FAS); Table S8: Unsolicited adverse events with frequency of ≥1% in any treatment group by System Organ Class and Preferred Term (FAS); Table S9: Institutional review boards and ethics committees; Table S10: E.ngage Investigators and Study Sites; Table S11: Independent Data Monitoring Committee members; Figure S1: ExPEC9V O-serotype MOPA antibody response on Day 1 and Day 30 (PPEI) and GMFI (95% CI) from baseline in ExPEC9V O-serotype MOPA-determined functional antibody; Figure S2: ExPEC9V O-serotype multiplex ECL-based immunoassay antibody responses on Day 1 and Day 30 (PPEI) and GMFI (95% CI) from baseline in ExPEC9V O-serotype total (IgG) antibody levels by history of UTI (Control group); Figure S3: ExPEC9V O-serotype MOPA antibody response on Day 1 and Day 30 (PPEI) and GMFI (95% CI) from baseline in ExPEC9V O-serotype MOPA-determined functional antibody by history of UTI (Control group).

## References

[1] Geurtsen J., de Been M., Weerdenburg E., Zomer A., McNally A., Poolman J. (2022). Genomics and pathotypes of the many faces of *Escherichia coli*. FEMS Microbiol. Rev..

[2] de Kraker M.E., Jarlier V., Monen J.C., Heuer O.E., van de Sande N., Grundmann H. (2013). The changing epidemiology of bacteraemias in Europe: Trends from the European Antimicrobial Resistance Surveillance System. Clin. Microbiol. Infect..

[3] Bonten M., Johnson J.R., van den Biggelaar A.H.J., Georgalis L., Geurtsen J., de Palacios P.I., Gravenstein S., Verstraeten T., Hermans P., Poolman J.T. (2021). Epidemiology of *Escherichia coli* bacteremia: A systematic literature review. Clin. Infect. Dis..

[4] Poolman J.T., Wacker M. (2016). Extraintestinal pathogenic *Escherichia coli*, a common human pathogen: Challenges for vaccine development and progress in the field. J. Infect. Dis..

[5] Rhee C., Kadri S.S., Dekker J.P., Danner R.L., Chen H.-C., Fram D., Zhang F., Wang R., Klompas M., for the CDC Prevention Epicenters Program (2020). Prevalence of antibiotic-resistant pathogens in culture-proven sepsis and outcomes associated with inadequate and broad-spectrum empiric antibiotic use. JAMA Netw. Open.

[6] Al-Hasan M.N., Lahr B.D., Eckel-Passow J.E., Baddour L.M. (2009). Antimicrobial resistance trends of *Escherichia coli* bloodstream isolates: A population-based study, 1998–2007. J. Antimicrob. Chemother..

[7] Al-Hasan M.N., Lahr B.D., Eckel-Passow J.E., Baddour L.M. (2009). Seasonal variation in *Escherichia coli* bloodstream infection: A population-based study. Clin. Microbiol. Infect..

[8] Huttner A., Hatz C., van den Dobbelsteen G., Abbanat D., Hornacek A., Frölich R., Dreyer A.M., Martin P., Davies T., Fae K. (2017). Safety, immunogenicity, and preliminary clinical efficacy of a vaccine against extraintestinal pathogenic *Escherichia coli* in women with a history of recurrent urinary tract infection: A randomised, single-blind, placebo-controlled phase 1b trial. Lancet Infect. Dis..

[9] Weerdenburg E., Davies T., Morrow B., Zomer A.L., Hermans P., Go O., Spiessens B., van den Hoven T., van Geet G., Aitabi M. (2023). Global distribution of O serotypes and antibiotic resistance in extraintestinal pathogenic *Escherichia coli* collected from the blood of patients with bacteremia across multiple surveillance studies. Clin. Infect. Dis..

[10] Fierro C.A., Sarnecki M., Doua J., Spiessens B., Go O., Davies T.A., van den Dobbelsteen G., Poolman J., Abbanat D., Haazen W. (2023). Safety, reactogenicity, immunogenicity, and dose selection of 10-valent extraintestinal pathogenic *Escherichia coli* bioconjugate vaccine (VAC52416) in adults aged 60–85 years in a randomized, multicenter, interventional, first-in-human, phase 1/2a study. Open Forum Infect. Dis..

[11] Fierro C.A., Sarnecki M., Spiessens B., Go O., Day T.A., Davies T.A., van den Dobbelsteen G., Poolman J., Abbanat D., Haazen W. (2024). A randomized phase 1/2a trial of ExPEC10V vaccine in adults with a history of UTI. npj Vaccines.

[12] Tan L., Trevas D., Falsey A.R. (2025). Adult vaccine coadministration is safe, effective, and acceptable: Results of a survey of the literature. Influenza Other Respir. Viruses.

[13] European Medicines Agency, Committee for Medicinal Products for Human Use (2023). Guideline on Clinical Evaluation of Vaccines.

[14] Sanofi Pasteur Inc Fluzone^®^ High-Dose Quadrivalent. Package Insert, Revised July 2025. https://www.fda.gov/media/132238/download.

[15] US Department of Health and Human Services, Food and Drug Administration, Center for Biologics Evaluation and Research (2007). Guidance for Industry: Toxicity Grading Scale for Healthy Adult and Adolescent Volunteers Enrolled in Preventive Vaccine Clinical Trials.

[16] Frenck R.W., Ervin J., Chu L., Abbanat D., Spiessens B., Go O., Haazen W., van den Dobbelsteen G., Poolman J., Thoelen S. (2019). Safety and immunogenicity of a vaccine for extra-intestinal pathogenic *Escherichia coli* (ESTELLA): A phase 2 randomised controlled trial. Lancet Infect. Dis..

[17] Inoue M., Ogawa T., Tamura H., Hagiwara Y., Saito Y., Abbanat D., van den Dobbelsteen G., Hermans P., Thoelen S., Poolman J. (2018). Safety, tolerability and immunogenicity of the ExPEC4V (JNJ-63871860) vaccine for prevention of invasive extraintestinal pathogenic *Escherichia coli* disease: A phase 1, randomized, double-blind, placebo-controlled study in healthy Japanese participants. Hum. Vaccin. Immunother..

[18] Severance R., Schwartz H., Dagan R., Connor L., Li J., Pedley A., Hartzel J., Sterling T.M., Nolan K.M., Tamms G.M. (2022). Safety, tolerability, and immunogenicity of V114, a 15-valent pneumococcal conjugate vaccine, administered concomitantly with influenza vaccine in healthy adults aged ≥ 50 years: A randomized phase 3 trial (PNEU-FLU). Hum. Vaccin. Immunother..

[19] Thompson A.R., Klein N.P., Downey H.J., Patterson S., Sundaraiyer V., Watson W., Clarke K., Jansen K.U., Sebastian S., Gruber W.C. (2019). Coadministration of 13-valent pneumococcal conjugate and quadrivalent inactivated influenza vaccines in adults previously immunized with polysaccharide pneumococcal vaccine 23: A randomized clinical trial. Hum. Vaccin. Immunother..

[20] Cannon K., Cardona J.F., Yacisin K., Thompson A., Belanger T.J., Lee D.Y., Peng Y., Moyer L., Ginis J., Gruber W.C. (2023). Safety and immunogenicity of a 20-valent pneumococcal conjugate vaccine coadministered with quadrivalent influenza vaccine: A phase 3 randomized trial. Vaccine.

[21] Schwarz T.F., Flamaing J., Rümke H.C., Penzes J., Juergens C., Wenz A., Jayawardene D., Giardina P., Emini E.A., Gruber W.C. (2011). A randomized, double-blind trial to evaluate immunogenicity and safety of 13-valent pneumococcal conjugate vaccine given concomitantly with trivalent influenza vaccine in adults aged ≥ 65 years. Vaccine.

