# Correction: Leroux-Roels et al. Immunogenicity and Safety of the ExPEC9V *Escherichia coli* Vaccine Co-Administered with a High-Dose Influenza Vaccine in Older Adults: A Placebo-Controlled, Randomized, Phase 3 Study. *Vaccines* 2026, *14*, 146

**DOI:** 10.3390/vaccines14080674

**Published:** 2026-08-04

**Authors:** Isabel Leroux-Roels, Tracey A. Day, Sofie Deleu, Chelsea McLean, Oscar Go, Todd A. Davies, Jeroen N. Stoop, Monika Peeters, Maria G. Pau, Bart Spiessens, Michal Sarnecki, Keira A. Cohen

**Affiliations:** 1 Center for Vaccinology, Ghent University and Ghent University Hospital, Corneel Heymanslaan 10, 9000 Ghent, Belgium; isabel.lerouxroels@uzgent.be; 2 Johnson & Johnson, Turnhoutseweg 30, 2340 Beerse, Belgium; tracey.day@ipsen.com (T.A.D.); sdeleu1@its.jnj.com (S.D.); mpeeter7@its.jnj.com (M.P.); bartspiessens0@gmail.com (B.S.); 3 Johnson & Johnson, Archimedesweg 4, 2333CN Leiden, The Netherlands; cmclean@its.jnj.com (C.M.); jstoop1@its.jnj.com (J.N.S.); mpau@its.jnj.com (M.G.P.); 4 Johnson & Johnson 920 US-202, Raritan, NJ 08869, USA; ogo@its.jnj.com (O.G.); tdavies1@its.jnj.com (T.A.D.); 5 Johnson & Johnson, Rehhagstrasse 79, 3018 Bern, Switzerland; sarnecki.m@recordati.com; 6 Johnson & Johnson, 1125 Bear Tavern Rd, New Brunswick, NJ 08560, USA

Acknowledgements

In the original publication [1], the names of some individuals were incorrectly included in the statement.

The corrected statement is “The authors thank all study participants, the E.ngage Study Team, investigators and study staff from all investigational sites (Table S10), members of the Independent Data Monitoring Committee (Table S11), and Tiziano de Rosa, Gloria Aguilar, Jyotsna Bhattacharya, Michael Katwere, Javier Ruiz-Guiñazú, Georgi Shukarev, Imre Lazlo, Valerie Oriol Mathieu, Jose Teixeira, Elizabeth Garcia Dominguez, and Sarah Tete van Erven from Johnson & Johnson and Beth Kelley from Sanofi for their contributions.”

Supplementary Materials

In the original publication, there were errors in the list of E.ngage Investigators and Study Sites (Table S10), and in the list of Independent Data Monitoring Committee members (Table S11).

The corrected tables are as follows:

**Table S10:** E.ngage Investigators and Study Sites.

Investigator	Primary Institution Name
**Belgium**	
Isabel Leroux-Roels	Center for Vaccinology,
Nikita Hanning	Universiteit Antwerpen—Centrum voor de Evaluatie van Vaccinaties
Muriel Lins	AZ Sint-Maarten
**Canada**	
Naresh Aggarwal	Aggarwal and associates Ltd.
Peter Dzongowski	Milestone Research
**Poland**	
Marek Konieczny	Velocity Lublin
Bozena Jachimczak	Pratia MCM Krakow
Marek Dwojak	Synexus Polska Sp z o o Oddzial we Wroclawiu
Malgorzata Sipinska-Surzynska	Synexus Polska Sp. z o.o. Oddzial w Katowicach
Monika Zaczek-Chmielewska	Synexus Polska Sp z o o Oddzial w Warszawie
Milena Kowalewska-Celejewska	Synexus Polska Sp. z o.o. Oddzial w Gdansku
**United States**	
Codey Bell	Tekton Research Inc.
Helen Stacey	Diablo Clinical Research, Inc.
Mark Adams	Alliance for Multispeciality Research
Jorge Caso	Suncoast Research Associates, LLC
David Fried	Velocity Clinical Research
Antoinette Pragalos	CTI Clinical Trial and Consulting Services
Margaret Rhee	Velocity Clinical Research
Robert Riesenberg	Atlanta Center for Medical Research
Murray Kimmel	Optimal Research
Terry Poling	Heartland Research Associates, an AMR Company

This list of investigators is not exhaustive, as it only includes individuals who have formally accepted acknowledgment.

**Table S11:** Independent Data Monitoring Committee members.

Expert	Committee
Oliver Cornely	IDMC
Isabel Leroux-Roels	IDMC
Geert Molenberghs	IDMC

This list of IDMC members is not exhaustive, as it only includes individuals who have formally accepted acknowledgment. IDMC, Independent Data Monitoring Committee.

The authors state that the scientific conclusions are unaffected. These corrections were approved by the Academic Editor. The original publication has also been updated.

## References

[1] Leroux-Roels I., Day T.A., Deleu S., McLean C., Go O., Davies T.A., Stoop J.N., Peeters M., Pau M.G., Spiessens B. (2026). Immunogenicity and Safety of the ExPEC9V *Escherichia coli* Vaccine Co-Administered with a High-Dose Influenza Vaccine in Older Adults: A Placebo-Controlled, Randomized, Phase 3 Study. Vaccines.

