# Supplementary material for: Immunogenicity and Safety of the ExPEC9V Escherichia coli Vaccine Co-Administered with a High-Dose Influenza Vaccine in Older Adults: A Placebo-Controlled, Randomized, Phase 3 Study
Source: Vaccines (Basel). 2026 Jan 30;14(2):146. doi: 10.3390/vaccines14020146 (PMC12944986; doi:10.3390/vaccines14020146)
Supplement: Supplementary file 1 [file vaccines-14-00146-s001.zip › vaccines-4085897-supplementary.pdf]

SUPPLEMENTARY MATERIAL

Supplementary Methods

Study Population

Inclusion Criteria

Each potential participant must satisfy all of the following criteria to be enrolled in the study:

Age
1. ≥65 years of age, inclusive, on the day of signing the informed consent form (ICF).

Type of Participant and Disease Characteristics
2. Must be medically stable at the time of vaccination such that, according to the judgment of the investigator, hospitalization within the study period was not anticipated and the participant appeared likely to be able to remain on study through the end of protocol-specified follow-up. A stable medical condition was defined as disease not requiring significant change in therapy during the 6 weeks before enrollment and when hospitalization for worsening of the disease was not anticipated. Participants were included on the basis of physical examination, medical history, and vital signs performed between ICF signature and vaccination.

Weight
Not applicable.

Sex and Contraceptive/Barrier Requirements
3. Male or female.
4. Before randomization, a participant must have been:
a. Postmenopausal (postmenopausal state was defined as no menses for 12 months without an alternative medical cause); and
b. Not intending to conceive by any methods.

Note: Surgically sterile participants were also eligible for the study.

Informed Consent
5. Must have signed an ICF indicating that the participant understood the purpose, procedures and potential risks and benefits of the study, and was willing to participate in the study.
6. Willing and able to adhere to the lifestyle restrictions specified in the protocol.

Additional Inclusion Criteria
7. Agreed to not donate blood from the time of vaccination until 3 months after receiving the last dose of study vaccine.
8. Was willing to provide verifiable identification, had means to be contacted and to contact the investigator during the study.
9. Was able to read, understand, and complete the eDiary.
10. Was able to work with smartphones/tablets/computers.

Exclusion Criteria

Any potential participant who met any of the following criteria were excluded from participating in the study:

Medical Conditions

1. History of an underlying clinically significant acute or uncontrolled chronic medical condition or significant cognitive impairment or physical examination findings for which, in the opinion of the investigator, participation would not be in the best interest of the participant (eg, compromise their well-being) or that could prevent, limit, or confound the protocol-specified assessments.

2. Abnormal function of the immune system resulting from:

a. clinical conditions or their treatments expected to have an impact on the immune response elicited by the study vaccine. Participants with clinical conditions that were stable under treatment without the use of prohibited therapies could be enrolled at the discretion of the investigator.

b. chronic or recurrent use of systemic corticosteroids within 3 months before administration of study vaccine and during the study. A substantially immunosuppressive steroid dose was considered to be ≥2 weeks of daily receipt of 20 mg of prednisone or equivalent.

Note: Ocular, topical or inhaled steroids were allowed.

c. administration of antineoplastic and immunomodulating agents (eg, cancer chemotherapeutic agents) or radiotherapy expected to have an impact on the immune response elicited by the study vaccine within 6 months before administration of study vaccine and during the study.

3. History of malignancy within 5 years before screening not in the following categories:

a. participants with squamous and basal cell carcinomas of the skin and carcinoma in situ of the cervix could be enrolled at the discretion of the investigator.

b. participants with a history of malignancy within 5 years before screening, with minimal risk of recurrence per investigator's judgment, could be enrolled.

c. participants with a diagnosis of localized prostate cancer could be enrolled at the discretion of the investigator if they completed treatment (continuation of androgen deprivation therapy was allowed) or if they remained under observation or active surveillance. Participants who underwent radical prostatectomy or radiotherapy could be enrolled at the discretion of the investigator if treatment had been completed 6 months prior to the planned administration of the study vaccine.

4. Known or suspected allergy or history of severe allergic reaction, anaphylaxis, or other serious adverse reactions to vaccines or vaccine excipients (specifically the excipients of the study vaccine).

5. History of severe allergic reactions (eg, anaphylaxis) to any component of the HD quadrivalent seasonal influenza vaccine, including egg protein, or following a previous dose of any influenza vaccine.

6. Had major surgery (per the investigator's judgment) within 4 weeks before administration of the first study vaccine or would not have recovered from surgery per the investigator's judgment at time of vaccination.

7. History of acute polyneuropathy (eg, Guillain–Barré syndrome) or chronic inflammatory demyelinating polyneuropathy.

8. Had major psychiatric illness or drug or alcohol abuse which in the investigator's opinion would compromise the participant's safety or compliance with the study procedures.
9. Contraindication to IM injections and blood draws (eg, bleeding disorders).

Prior/Concomitant Therapy

10. Received hematopoietic stem cell transplant based on medical history, treatment with immunoglobulins in the 2 months, apheresis therapies in the 4 months, or blood products in the 3 months before the planned administration of the first dose of study vaccine or had any plans to receive such treatment during the study.

> ***Note**: Given that not all immunoglobulins/monoclonal antibodies were expected to impact the vaccine-induced immune response, the investigator was to contact the sponsor to discuss eligibility of participants on immunoglobulin treatment.*

11. Received or planned to receive:
a. licensed live attenuated vaccines—within 28 days before or after planned administration of the first or subsequent study vaccinations.
b. Other licensed (not live) vaccines—within 14 days before or after planned administration of the first or subsequent study vaccinations.
c. Vaccination with a vaccine authorized for emergency use (eg, EUA, CMA, or a similar program) was permitted when given at least 28 days before or after planned administration of the first or subsequent study vaccinations.
12. Received vaccination with seasonal influenza vaccine for the current influenza season in the Northern Hemisphere.
13. Received any *E. coli* or ExPEC vaccine.

Prior/Concurrent Clinical Study Experience

14. Received an investigational drug or used an invasive investigational medical device within 90 days, or received an investigational vaccine within 90 days before the planned administration of the first dose of study vaccine, or was currently enrolled or planned to participate in another investigational study during the course of this study and before 6 months after administration of the study vaccine.

Diagnostic Assessments

15. Had uncontrolled HIV type 1 or type 2 infection.

> ***Note**: a participant with a stable/well-controlled HIV infection was allowed.*

16. Had a diagnosis of chronic active hepatitis B or hepatitis C infection that was not medically stable, based on judgment of the investigator.

> ***Note**: a participant with a stable and virologically suppressed hepatitis B or hepatitis C infection was allowed.*

Other Exclusions

17. Employee of the investigator or study site with direct involvement in the proposed study or other studies under the direction of that investigator or study site, as well as family members of the employees or the investigator, or an employee of the sponsor or CRO.
18. Could not communicate reliably with the investigator.
19. Who, in the opinion of the investigator, was unlikely to adhere to the requirements of the study, or was unlikely to complete the full course of vaccination and observation.

20. Who had significant scarring, tattoos, abrasions, cuts, or infections over the deltoid region of both arms that, in the investigator's opinion, could interfere with evaluation of injection site local reactions.

> ***Note:*** *Investigators were to ensure that all study enrollment criteria had been met prior to the first vaccination. If a participant's clinical status changed (including any available laboratory results or receipt of additional medical records) after screening but before the first study vaccination was given such that the participant no longer met all eligibility criteria, then the participant was excluded from participation in the study.*

Table S1. HAI antibody response to influenza strains: Comparison between groups (Control / CoAd) using age and history of UTI as collected (PPII).

Influenza Strain	LS Means (95% CI)[a]		
	CoAd	Control	GMR (95% CI)[b]
A/Victoria [H1N1]	368.94 (321.31–423.62)	362.43 (316.39–415.16)	0.982 (0.832–1.160)
A/Darwin [H3N2]	328.38 (280.72–384.13)	335.91 (287.93–391.89)	1.023 (0.847–1.235)
B/Victoria	727.27 (647.29–817.12)	703.30 (627.21–788.62)	0.967 (0.841–1.113)
B/Yamagata	638.64 (572.77–712.07)	572.60 (514.50–637.26)	0.897 (0.787–1.022)

[a] LS means and corresponding CI of the log-transformed HAI antibody titers, back-transformed (by exponentiation) to a GMT.

[b]An ANOVA model was fitted (using SAS proc mixed) per influenza vaccine strain, with the respective Day 30 titers (log10-transformed) as dependent variable, and group (Control or CoAd), age category (as collected), and history of UTI (as collected) as independent variables. Based on these ANOVA models, the CIs around the difference (Control group minus CoAd group) was calculated and back-transformed (by exponentiation: 10^CI) to CIs around a GMR ($GMT_{Control}/GMT_{CoAd}$).

CoAd: ExPEC9V + HD influenza vaccine (Day 1), Placebo (Day 30).

Control: Placebo + HD influenza vaccine (Day 1), ExPEC9V (Day 30).

ANOVA = analysis of variance, CI = confidence interval, CoAd = co-administration, ExPEC9V = 9-valent extraintestinal pathogenic *Escherichia coli* vaccine, GMR = geometric mean ratio, GMT = geometric mean titer, HAI = hemagglutination inhibition, HD = high-dose, A/Victoria [H1N1])= A/Victoria/4897/2022 IVR-238, A/Darwin [H3N2] = A/Darwin/9/2021, B/Victoria = B/Michigan/1/2021, B/Yamagata = B/Phuket/3073/2013, LS = least squares, PPII = Per-Protocol Influenza Immunogenicity set, UTI = urinary tract infection.

Table S2. Number and percentage of participants with a ≥2-fold increase in total (IgG) antibody levels 29 days post vaccination with ExPEC9V (PPEI).

Serotype	CoAd (*n*/*N* = 448/466)	Control (*n*/*N* = 442/464)
O1A		
n (%)	389 (86.8)	394 (89.1)
(95% CI)	(83.34–89.82)	(85.86–91.88)
O2		
n (%)	410 (91.5)	418 (94.6)
(95% CI)	(88.54–93.93)	(92.03–96.49)
O4		
n (%)	287 (64.1)	340 (76.9)
(95% CI)	(59.43–68.51)	(72.71–80.77)
O6A		
n (%)	310 (69.2)	355 (80.3)
(95% CI)	(64.69–73.44)	(76.30–83.92)
O15		
n (%)	333 (74.3)	365 (82.6)
(95% CI)	(70.02–78.31)	(78.72–86.00)
O16		
n (%)	360 (80.4)	397 (89.8)
(95% CI)	(76.37–83.94)	(86.61–92.48)
O18A		
n (%)	361 (80.6)	387 (87.6)
(95% CI)	(76.61–84.14)	(84.11–90.49)
O25B		
n (%)	347 (77.5)	389 (88.0)
(95% CI)	(73.30–81.24)	(84.61–90.89)
O75		
n (%)	277 (61.8)	301 (68.1)
(95% CI)	(57.15–66.35)	(63.53–72.42)
EPA (ECL only)		
n (%)	390 (87.1)	399 (90.3)
(95% CI)	(83.59–90.02)	(87.12–92.87)

n, number of participants with a ≥2-fold increase.
Day is shown relative to ExPEC9V vaccination.
95% CI is based on Clopper–Pearson method.
CoAd: ExPEC9V + HD influenza vaccine (Day 1), placebo (Day 30). Control: placebo + HD influenza vaccine (Day 1), ExPEC9V (Day 30).
CI = confidence interval, CoAd = co-administration, ECL = electrochemiluminescence, EPA = ExoProtein A, ExPEC9V = 9-valent extraintestinal pathogenic *Escherichia coli* vaccine, HD = high-dose, PPEI = Per-Protocol ExPEC9V Immunogenicity set,.

Table S3. O-Serotype MOPA antibody response (PPEI).

	LS Means (95% CI)[a]		
**O-Serotype**	**CoAd**	**Control**	**GMR (95% CI)[b]**
O1A	1587.20 (1336.33–1885.16)	2093.03 (1764.40–2482.86)	1.319 (1.071–1.624)
O2	2219.85 (1844.81–2671.14)	3418.07 (2844.25–4107.66)	1.540 (1.231–1.927)
O4	415.08 (360.08–478.49)	506.64 (439.96–583.43)	1.221 (1.028–1.450)
O6A	1153.71 (986.62–1349.08)	1303.77 (1116.21–1522.83)	1.130 (0.935–1.366)
O15	1320.84 (1125.98–1549.42)	1783.45 (1506.49–2111.31)	1.350 (1.107–1.647)
O16	1105.31 (917.77–1331.16)	1784.20 (1484.73–2144.07)	1.614 (1.289–2.021)
O18A	617.60 (532.47–716.35)	1029.51 (867.94–1221.17)	1.667 (1.371–2.026)
O25B	128.06 (109.68–149.52)	175.37 (150.31–204.62)	1.369 (1.135–1.652)
O75	92.21 (77.73–109.38)	159.09 (132.13–191.54)	1.725 (1.392–2.139)

[a]LS means and corresponding CI of the log-transformed ExPEC9V O-serotypes titers, back-transformed (by exponentiation) to a GMT.

[b]An ANOVA model was fitted (using SAS proc mixed) with the log10-transformed Day 30 (relative to ExPEC vaccination) ExPEC9V O-serotypes titers as dependent variable, and group (Control or CoAd), age category (as collected), history of UTI (as collected) as independent variables. Based on this ANOVA model, the CIs around the difference (Control group minus CoAd group) was calculated and back-transformed (by exponentiation: 10^CI) to CIs around a GMR ($GMT_{Control}$/GMT_{CoAd}).

CoAd: ExPEC9V + HD influenza vaccine (Day 1), Placebo (Day 30).

Control: Placebo + HD influenza vaccine (Day 1), ExPEC9V (Day 30).

ANOVA = analysis of variance, CI = confidence interval, CoAd = co-administration, ExPEC9V = 9-valent extraintestinal pathogenic *Escherichia coli* vaccine, GMR = geometric mean ratio, GMT = geometric mean titer, HD = high-dose, LS = least squares, MOPA = multiplex opsonophagocytic assay, PPEI = Per-Protocol ExPEC9V Immunogenicity set, UTI = urinary tract infection.

Table S4. Number and percentage of participants with a ≥2-fold increase in total (IgG) antibody levels 29 days post vaccination with ExPEC9V by ECL by history of UTI (PPEI).

	History of UTI at baseline		No history of UTI at baseline	
**Serotype**	**CoAd (*N* = 113)**	**Control (*N* = 120)**	**CoAd (*N* = 353)**	**Control (*N* = 344)**
Number of participants with data, *N*	109	114	339	328
O1A				
n (%)	99 (90.8)	102 (89.5)	290 (85.5)	292 (89.0)
(95% CI)	(83.77–95.51)	(82.33–94.44)	(81.34–89.11)	(85.13–92.19)
O2				
n (%)	98 (89.9)	111 (97.4)	312 (92.0)	307 (93.6)
(95% CI)	(82.66–94.85)	(92.50–99.45)	(88.62–94.69)	(90.38–95.99)
O4				
n (%)	65 (59.6)	91 (79.8)	222 (65.5)	249 (75.9)
(95% CI)	(49.81–68.92)	(71.28–86.76)	(60.16–70.54)	(70.91–80.44)
O6A				
n (%)	75 (68.8)	95 (83.3)	235 (69.3)	260 (79.3)
(95% CI)	(59.22–77.34)	(75.20–89.66)	(64.11–74.19)	(74.47–83.53)
O15				
n (%)	78 (71.6)	92 (80.7)	255 (75.2)	273 (83.2)
(95% CI)	(62.12–79.79)	(72.25–87.49)	(70.27–79.73)	(78.74–87.11)
O16				
n (%)	89 (81.7)	103 (90.4)	271 (79.9)	294 (89.6)
(95% CI)	(73.09–88.42)	(83.39–95.08)	(75.28–84.07)	(85.82–92.71)
O18A				
n (%)	91 (83.5)	105 (92.1)	270 (79.6)	282 (86.0)
(95% CI)	(75.16–89.91)	(85.54–96.33)	(74.96–83.80)	(81.74–89.55)
O25B				
n (%)	86 (78.9)	97 (85.1)	261 (77.0)	292 (89.0)
(95% CI)	(70.04–86.13)	(77.20–91.07)	(72.14–81.37)	(85.13–92.19)
O75				
n (%)	63 (57.8)	81 (71.1)	214 (63.1)	220 (67.1)
(95% CI)	(47.96–67.20)	(61.81–79.16)	(57.75–68.28)	(61.70–72.14)
EPA (ECL only)				
n (%)	95 (87.2)	105 (92.1)	295 (87.0)	294 (89.6)
(95% CI)	(79.39–92.80)	(85.54–96.33)	(82.97–90.41)	(85.82–92.71)

n, number of participants with a ≥2-fold increase.
Day is shown relative to ExPEC vaccination.
Number of participants with data: CoAd, *n* = 448; Control, *n* = 442.
95% CI is based on Clopper–Pearson method.
CoAd: ExPEC9V + HD influenza vaccine (Day 1), placebo (Day 30). Control: placebo + HD influenza vaccine (Day 1), ExPEC9V (Day 30).
CI = confidence interval, CoAd = co-administration, ECL = electrochemiluminescence, EPA = ExoProtein A, ExPEC9V = 9-valent extraintestinal pathogenic *Escherichia coli* vaccine, HD = high-dose, PPEI = Per-Protocol

ExPEC9V Immunogenicity set, UTI = urinary tract infection.

Table S5. Solicited local adverse events by derived term and worst severity (FAS).

	Post Vaccination 1				Post Vaccination 2	
	ExPEC9V + HD Influenza Vaccine (CoAd)		Placebo + HD Influenza Vaccine (Control)		Placebo (CoAd)	ExPEC9V (Control)
**AE Type, *n* (%)**	**ExPEC9V Injection Site (*N* = 476)**	**HD Influenza Vaccines Injection Site (*N* = 476)**	**Placebo Injection Site (*N* = 481)**	**HD Influenza Vaccine Injection Site (*N* = 481)**	**Placebo Injection Site (*N* = 461)**	**ExPEC9V Injection Site (*N* = 466)**
Participants with ≥1 local AE						
Any	203 (42.6)	218 (45.8)	94 (19.5)	231 (48.0)	55 (11.9)	164 (35.2)
Grade 1	132 (27.7)	188 (39.5)	87 (18.1)	208 (43.2)	49 (10.6)	103 (22.1)
Grade 2	56 (11.8)	27 (5.7)	7 (1.5)	23 (4.8)	5 (1.1)	51 (10.9)
Grade 3	15 (3.2)	3 (0.6)	0	0	1 (0.2)	10 (2.1)
Vaccination site erythema						
Any	64 (13.4)	19 (4.0)	9 (1.9)	26 (5.4)	5 (1.1)	58 (12.4)
Grade 1	27 (5.7)	12 (2.5)	7 (1.5)	19 (4.0)	4 (0.9)	23 (4.9)
Grade 2	23 (4.8)	6 (1.3)	2 (0.4)	7 (1.5)	1 (0.2)	27 (5.8)
Grade 3	14 (2.9)	1 (0.2)	0	0	0	7 (1.5)
Vaccination site pain						
Any	198 (41.6)	213 (44.7)	83 (17.3)	225 (46.8)	53 (11.5)	160 (34.3)
Grade 1	145 (30.5)	193 (40.5)	79 (16.4)	209 (43.5)	49 (10.6)	118 (25.3)
Grade 2	50 (10.5)	18 (3.8)	4 (0.8)	16 (3.3)	3 (0.7)	39 (8.4)
Grade 3	3 (0.6)	2 (0.4)	0	0	1 (0.2)	3 (0.6)
Vaccination site swelling						
Any	47 (9.9)	27 (5.7)	8 (1.7)	29 (6.0)	2 (0.4)	47 (10.1)
Grade 1	24 (5.0)	18 (3.8)	7 (1.5)	24 (5.0)	1 (0.2)	25 (5.4)
Grade 2	20 (4.2)	9 (1.9)	1 (0.2)	5 (1.0)	1 (0.2)	19 (4.1)
Grade 3	3 (0.6)	0	0	0	0	3 (0.6)

Participants are counted only once for any given event, regardless of the number of times they actually experienced the event.
The event experienced by the participant with the worst toxicity grade is used. If a participant has missing toxicity grade for a specific AE, the participant is counted in the "Any" row for that AE.
CoAd: ExPEC9V + HD influenza vaccine (Day 1), Placebo (Day 30).
Control: Placebo + HD influenza vaccine (Day 1), ExPEC9V (Day 30).
Note: For Post-dose 1, if a participant received their vaccinations in the same arm, the solicited local AE was counted for both the ExPEC9V (or placebo) injection site column and the HD influenza vaccine injection site column.
AE = adverse event, CoAd = co-administration, ExPEC9V = 9-valent extraintestinal pathogenic *Escherichia coli* vaccine, FAS = full analysis set, HD = high-dose.

Table S6. Duration and timing of solicited local adverse events by derived term (FAS).

	Post Vaccination 1				Post Vaccination 2	
	ExPEC9V + HD Influenza Vaccine (CoAd)		Placebo + HD Influenza Vaccine (Control)		Placebo (CoAd)	ExPEC9V (Control)
**AE Type**	**ExPEC9V Injection Site (*N* = 476)**	**HD Influenza Vaccine Injection Site (*N* = 476)**	**Placebo Injection Site (*N* = 481)**	**HD Influenza Vaccine Injection Site (*N* = 481)**	**Placebo Injection Site (*N* = 461)**	**ExPEC9V Injection Site (*N* = 466)**
Participants with ≥1 solicited local AE	203 (42.6)	218 (45.8)	94 (19.5)	231 (48.0)	55 (11.9)	164 (35.2)
Early onset	90 (18.9)	210 (44.1)	82 (17.0)	222 (46.2)	45 (9.8)	42 (9.0)
Late onset	113 (23.7)	8 (1.7)	12 (2.5)	9 (1.9)	10 (2.2)	122 (26.2)
Vaccination Site Erythema	64 (13.4)	19 (4.0)	9 (1.9)	26 (5.4)	5 (1.1)	58 (12.4)
Median time to onset (min, max), days	8.0 (1,11)	2.0 (1,9)	3.0 (1,11)	2.0 (1,14)	1.0 (1,2)	8.0 (1,11)
Median duration (min, max), days	3.0 (1,13)	2.0 (1,8)	1.0 (1,13)	3.0 (1,13)	1.0 (1,2)	2.0 (1,8)
Early onset	8 (1.7)	18 (3.8)	7 (1.5)	25 (5.2)	5 (1.1)	3 (0.6)
Late onset	26 (11.8)	1 (0.2)	2 (0.4)	1 (0.2)	0	55 (11.8)
Vaccination Site Pain	198 (41.6)	213 (44.7)	83 (17.3)	225 (46.8)	53 (11.5)	160 (34.3)
Median time to onset (min, max), days	6.0 (1,11)	1.0 (1,13)	2.0 (1,14)	1.0 (1,14)	2.0 (1,13)	7.0 (1,12)
Median duration (min, max), days	3.0 (1,14)	2.0 (1,13)	1.0 (1,14)	2.0 (1,32)	2.0 (1,13)	2.0 (1,14)
Early onset	86 (18.1)	206 (43.3)	72 (15.0)	217 (45.1)	43 (9.3)	40 (8.6)
Late onset	112 (23.5)	7 (1.5)	11 (2.3)	8 (1.7)	10 (2.2)	120 (25.8)
Vaccination Site Swelling	47 (9.9)	27 (5.7)	8 (1.7)	29 (6.0)	2 (0.4)	47 (10.1)
Median time to onset (min, max), days	7.0 (1,11)	2.0 (1,9)	3.0 (2,10)	2.0 (1, 9)	2.0 (1,3)	8.0 (3,11)

AE Type	Post Vaccination 1				Post Vaccination 2	
	**ExPEC9V + HD Influenza Vaccine (CoAd)**		**Placebo + HD Influenza Vaccine (Control)**		**Placebo (CoAd)**	**ExPEC9V (Control)**
	**ExPEC9V Injection Site (*N* = 476)**	**HD Influenza Vaccine Injection Site (*N* = 476)**	**Placebo Injection Site (*N* = 481)**	**HD Influenza Vaccine Injection Site (*N* = 481)**	**Placebo Injection Site (*N* = 461)**	**ExPEC9V Injection Site (*N* = 466)**
Median duration (min, max), days	3.0 (1,11)	1.0 (1,10)	1.5 (1,13)	1.0 (1,14)	6.0 (2,10)	2.0 (1,6)
Early onset	12 (2.5)	26 (5.5)	7 (1.5)	28 (5.8)	2 (0.4)	1 (0.2)
Late onset	35 (7.4)	1 (0.2)	1 (0.2)	1 (0.2)	0	46 (9.9)

Data are *n* (%), except where noted.
Early onset: Time to first onset of solicited AE is early if the solicited AE occurred on or before Day 5. Late onset: Time to first onset of solicited AE is late if the solicited AE occurred after Day 5.
CoAd: ExPEC9V + HD influenza vaccine (Day 1), Placebo (Day 30).
Control: Placebo + HD influenza vaccine (Day 1), ExPEC9V (Day 30).
Note: For Post-dose 1, if a participant received their vaccinations in the same arm, the solicited local AE was counted for both the ExPEC9V (or Placebo) injection site column and the HD influenza vaccine injection site column.
Duration is calculated by end date - start date + 1. Duration is measured by considering the maximum consecutive days of the occurrence of the event.
AEs occurring both early and late were included in each AE occurrence time period.
AE = adverse event, CoAd = co-administration, ExPEC9V = 9-valent extraintestinal pathogenic *Escherichia coli* vaccine, FAS = full analysis set, HD = high-dose.

Table S7. Solicited systemic adverse events by derived term and worst severity grade (FAS).

	Post Vaccination 1		Post Vaccination 2	
AE Type, *n* (%)	ExPEC9V + HD Influenza Vaccine (CoAd) (*N* = 476)	Placebo + HD Influenza Vaccine (Control) (*N* = 481)	Placebo (CoAd) (*N* = 461)	ExPEC9V (Control) (*N* = 466)
Solicited systemic AE				
Any	256 (53.8)	216 (44.9)	120 (26.0)	172 (36.9)
Grade 1	191 (40.1)	175 (36.4)	104 (22.6)	122 (26.2)
Grade 2	57 (12.0)	27 (5.6)	15 (3.3)	40 (8.6)
Grade 3	8 (1.7)	14 (2.9)	1 (0.2)	10 (2.1)
Fatigue				
Any	184 (38.7)	135 (28.1)	77 (16.7)	111 (23.8)
Grade 1	147 (30.9)	111 (23.1)	69 (15.0)	76 (16.3)
Grade 2	34 (7.1)	21 (4.4)	7 (1.5)	33 (7.1)
Grade 3	3 (0.6)	3 (0.6)	1 (0.2)	2 (0.4)
Headache				
Any	139 (29.2)	119 (24.7)	60 (13.0)	95 (20.4)
Grade 1	110 (23.1)	97 (20.2)	53 (11.5)	78 (16.7)
Grade 2	28 (5.9)	17 (3.5)	6 (1.3)	15 (3.2)
Grade 3	1 (0.2)	5 (1.0)	1 (0.2)	2 (0.4)
Myalgia				
Any	154 (32.4)	131 (27.2)	56 (12.1)	105 (22.5)
Grade 1	127 (26.7)	116 (24.1)	52 (11.3)	83 (17.8)
Grade 2	24 (5.0)	11 (2.3)	3 (0.7)	18 (3.9)
Grade 3	3 (0.6)	4 (0.8)	1 (0.2)	4 (0.9)
Nausea				
Any	66 (13.9)	38 (7.9)	30 (6.5)	35 (7.5)

AE Type, *n* (%)	Post Vaccination 1		Post Vaccination 2	
	**ExPEC9V + HD Influenza Vaccine (CoAd) (*N* = 476)**	**Placebo + HD Influenza Vaccine (Control) (*N* = 481)**	**Placebo (CoAd) (*N* = 461)**	**ExPEC9V (Control) (*N* = 466)**
Grade 1	56 (11.8)	31 (6.4)	26 (5.6)	28 (6.0)
Grade 2	8 (1.7)	3 (0.6)	4 (0.9)	7 (1.5)
Grade 3	2 (0.4)	4 (0.8)	0	0
Pyrexia				
Any	20 (4.2)	11 (2.3)	5 (1.1)	18 (3.9)
Grade 1	13 (2.7)	6 (1.2)	4 (0.9)	13 (2.8)
Grade 2	6 (1.3)	1 (0.2)	1 (0.2)	3 (0.6)
Grade 3	1 (0.2)	4 (0.8)	0	2 (0.4)

Participants are counted only once for any given event, regardless of the number of times they actually experienced the event.
The event experienced by the participant with the worst toxicity grade is used. If a participant has missing toxicity grade for a specific AE, the participant is counted in the "Any" row for that AE.
CoAd: ExPEC9V + HD influenza vaccine (Day 1), Placebo (Day 30).
Control: Placebo + HD influenza vaccine (Day 1), ExPEC9V (Day 30).
AE = adverse event, CoAd = co-administration, ExPEC9V = 9-valent extraintestinal pathogenic *Escherichia coli* vaccine, FAS = full analysis set, HD, high-dose.

Table S8. Unsolicited adverse events with frequency of ≥1% in any treatment group by System Organ Class and Preferred Term (FAS).

AE Type, *n* (%)	Post Vaccination 1		Post Vaccination 2	
	**ExPEC9V + HD Influenza Vaccine (CoAd) (*N* = 476)**	**Placebo + HD Influenza Vaccine (Control) (*N* = 481)**	**Placebo (CoAd) (*N* = 461)**	**ExPEC9V (Control) (*N* = 466)**
Any unsolicited AE	73 (15.3)	85 (17.7)	62 (13.4)	70 (15.0)
Infections and infestations	33 (6.9)	36 (7.5)	30 (6.5)	29 (6.2)
Nasopharyngitis	7 (1.5)	3 (0.6)	10 (2.2)	7 (1.5)
Upper respiratory tract infection	6 (1.3)	7 (1.5)	5 (1.1)	9 (1.9)
COVID-19	6 (1.3)	10 (2.1)	5 (1.1)	1 (0.2)
Urinary tract infection	5 (1.1)	4 (0.8)	3 (0.7)	5 (1.1)
Gastrointestinal disorders	8 (1.7)	8 (1.7)	8 (1.7)	6 (1.3)
Diarrhea	5 (1.1)	2 (0.4)	1 (0.2)	0

Participants are counted only once for any given event, regardless of the number of times they actually experienced the event.
AEs are coded using MedDRA Version 26.1.
CoAd: ExPEC9V + HD influenza vaccine (Day 1), Placebo (Day 30).
Control: Placebo + HD influenza vaccine (Day 1), ExPEC9V (Day 30).
AE = adverse event, CoAd = co-administration, ExPEC9V = 9-valent extraintestinal pathogenic *Escherichia coli* vaccine, FAS = full analysis set, HD = high-dose, MedDRA = Medical Dictionary for Regulatory Activities.

Table S9. Institutional review boards and ethics committees.

IRB/EC Name/Address	Site	Approval date
**BELGIUM**		
EU CTR EC - Domenico Scarlattilaan 6, Amsterdam, 1083 HS, Netherlands	BJ8-BE10001<br>University Hospital, Building VC001 - Ingang 99 - Route 995, Corneel Heymanslaan 10, Gent, 9000, Belgium	Region: Sep 18, 2023
EU CTR EC - Domenico Scarlattilaan 6, Amsterdam, 1083 HS, Netherlands	BJ8-BE10002<br>Drie Eikenstraat 663, First floor, Edegem, 2650, Belgium	Region: Sep 18, 2023
EU CTR EC - Domenico Scarlattilaan 6, Amsterdam, 1083 HS, Netherlands	BJ8-BE10003<br>Liersesteenweg 435, Mechelen, 2800, Belgium	Region: Sep 18, 2023
**CANADA**		
Veritas IRB Inc., 3551 St. Charles Blvd, Suite 501, Kirkland, QC, H9H 3C4	BJ8-CA10001<br>490 Bramalea Road, Suite 201, Brampton, ON L6T 0G1	Site, Central: Sep 26, 2023
Veritas IRB Inc., 3551 St. Charles Blvd, Suite 501, Kirkland, QC, H9H 3C4	BJ8-CA10002<br>295 Saskatoon Street, London, ON, N5W 6A2	Site, Central: Sep 19, 2023
Veritas IRB Inc., 3551 St. Charles Blvd, Suite 501, Kirkland, QC, H9H 3C4	BJ8-CA10006<br>1190A, Rue de Courchevel, Local 203, Levis, QC, G6W 0M5	Site, Central: Sep 28, 2023
**POLAND**		
EU CTR EC - Domenico Scarlattilaan 6, Amsterdam, 1083 HS, Netherlands	BJ8-PL10002<br>ul. Kochanowskiego 114, Gdansk, 80405	Site, Central: Sep 26, 2023
EU CTR EC - Domenico Scarlattilaan 6, Amsterdam, 1083 HS, Netherlands	BJ8-PL10004<br>ul K Przerwy Tetmajera 21, Lublin, Lubelskie, 20 362	Site, Central: Sep 26, 2023
EU CTR EC - Domenico Scarlattilaan 6, Amsterdam, 1083 HS, Netherlands	BJ8-PL10005<br>ul Pana Tadeusza 2, Krakow, 30 727	Site, Central: Sep 26, 2023
EU CTR EC - Domenico Scarlattilaan 6, Amsterdam, 1083 HS, Netherlands	BJ8-PL10006<br>Ul Marii Sklodowskiej Curie 12, Wrocław, 50 381	Site, Central: Sep 26, 2023
EU CTR EC - Domenico Scarlattilaan 6, Amsterdam, 1083 HS, Netherlands	BJ8-PL10007<br>ul. Konckiego 3, Oddzial Katowice, Katowice, 40-040	Site, Central: Sep 26, 2023
EU CTR EC - Domenico Scarlattilaan 6, Amsterdam, 1083 HS, Netherlands	BJ8-PL10008<br>Ul Domaniewska 49, Warszawam, 02 672	Site, Central: Sep 26, 2023
EU CTR EC - Domenico Scarlattilaan 6, Amsterdam, 1083 HS, Netherlands	BJ8-PL10009<br>Ul Beniowskiego 23, Gdansk, 80 382	Site, Central: Sep 26, 2023
EU CTR EC - Domenico Scarlattilaan 6, Amsterdam, 1083 HS, Netherlands	BJ8-PL10010<br>Ul Wronia 53 lok B10, Warszawa, 00 874	Site, Central: Sep 26, 2023
EU CTR EC - Domenico Scarlattilaan 6, Amsterdam, 1083 HS, Netherlands	BJ8-PL10011<br>ul Batorego 18-22, Torun, 87-100	Site, Central: Sep 26, 2023
EU CTR EC - Domenico Scarlattilaan 6, Amsterdam, 1083 HS, Netherlands	BJ8-PL10013<br>ul 11 Listopada 78, Staszow, 28 200	Site, Central: Sep 26, 2023
EU CTR EC - Domenico Scarlattilaan 6, Amsterdam, 1083 HS, Netherlands	BJ8-PL10015<br>ul. Luzycka 3c, Gdynia, Pomorskie, 81-537	Site, Central: Sep 26, 2023
**UNITED STATES**		
Sterling IRB, 5500 Interstate North Parkway, Suite 515, Atlanta, GA, 30328	BJ8-US10002	Site, Central: Jun 22, 2023

IRB/EC Name/Address	Site	Approval date
	100 Memorial Hospital Drive, Mobile, AL, 36608	
Sterling IRB, 5500 Interstate North Parkway, Suite 515, Atlanta, GA, 30328	BJ8-US10003<br>3560 Delaware Street, Suite 105, Beaumont, TX, 77706	Site, Central: Jul 13, 2023
Sterling IRB, 5500 Interstate North Parkway, Suite 515, Atlanta, GA, 30328	BJ8-US10005<br>1907 Tradd Ct, Wilmington, NC, 28401	Site, Central: Aug 03, 2023
Sterling IRB, 5500 Interstate North Parkway, Suite 515, Atlanta, GA, 30328	BJ8-US10007<br>2255 Ygnacio Valley Road, Suite M, Walnut Creek, CA, 94598	Site, Central: Jul 24, 2023
Sterling IRB, 5500 Interstate North Parkway, Suite 515,<br>Atlanta, GA, 30328	BJ8-US10009<br>303 Williams Avenue SW, Suite 511, Huntsville, AL, 35801	Site, Central: Sep 12, 2023
Sterling IRB, 5500 Interstate North Parkway, Suite 515, Atlanta, GA, 30328	BJ8-US10010<br>3475 Richmond Road, Lexington, KY, 40509	Site, Central: Jun 21, 2023
Sterling IRB, 5500 Interstate North Parkway, Suite 515, Atlanta, GA, 30328	BJ8-US10011<br>3100 Red River Street, Austin, TX, 78705	Site, Central: Aug 01, 2023
Sterling IRB, 5500 Interstate North Parkway, Suite 515, Atlanta, GA, 30328	BJ8-US10013<br>4061 West 95th Street, Oak Lawn, IL, 60453	Site, Central: Oct 25, 2023
Sterling IRB, 5500 Interstate North Parkway, Suite 515, Atlanta, GA, 30328	BJ8-US10014<br>340 Eisenhower Drive Oakdale, Savannah, GA, 31406	Site, Central: Aug 17, 2023
Sterling IRB, 5500 Interstate North Parkway, Suite 515, Atlanta, GA, 30328	BJ8-US10015<br>9260 Sunset Drive, Suite 107, Miami, FL, 33173	Site, Central: Jul 07, 2023
Sterling IRB, 5500 Interstate North Parkway, Suite 515, Atlanta, GA, 30328	BJ8-US10016<br>700 Medical Center Drive, Suite 110, Newton, KS, 67114	Site, Central: Jun 21, 2023
Sterling IRB, 5500 Interstate North Parkway, Suite 515, Atlanta, GA, 30328	BJ8-US10018<br>7200 NW 7th Street, Suite 350, Miami, FL, 33126	Site, Central: Aug 03, 2023
Sterling IRB, 5500 Interstate North Parkway, Suite 515, Atlanta, GA, 30328	BJ8-US10019<br>770 Washington St., Suite 101, San Diego, CA, 92103	Site, Central: Jul 10, 2023
Sterling IRB, 5500 Interstate North Parkway, Suite 515, Atlanta, GA, 30328	BJ8-US10020<br>1598 South County Trail, Suite 204, East Greenwich, RI, 02818	Site, Central: Aug 17, 2023
Sterling IRB, 5500 Interstate North Parkway, Suite 515, Atlanta, GA, 30328	BJ8-US10021<br>1775 Lexington Avenue, Cincinnati, OH, 45212	Site, Central: Jul 13, 2023
Sterling IRB, 5500 Interstate North Parkway, Suite 515, Atlanta, GA, 30328	BJ8-US10022<br>3619 Park East Dr. Suite 300, Cleveland, OH, 44122	Site, Central: Sep 15, 2023
Sterling IRB, 5500 Interstate North Parkway, Suite 515, Atlanta, GA, 30328	BJ8-US10023<br>501 Fairburn Rd, SW, Atlanta, GA, 30331	Site, Central: Aug 03, 2023
Sterling IRB, 5500 Interstate North Parkway, Suite 515, Atlanta, GA, 30328	BJ8-US10025<br>801 N. Weisgarber Road Suite 100, Knoxville, TN, 37909	Site, Central: Jun 16, 2023
Sterling IRB, 5500 Interstate North Parkway, Suite 515, Atlanta, GA, 30328	BJ8-US10026	Site, Central: Aug 08, 2023

IRB/EC Name/Address	Site	Approval date
	7551 Metro Center Drive, Suite(s) 200 and 400, Austin, TX, 78744	
Sterling IRB, 5500 Interstate North Parkway, Suite 515, Atlanta, GA, 30328	BJ8-US10027<br>3030 Venture Lane, Suite 101, Melbourne, FL, 32934	Site, Central: Aug 04, 2023
Sterling IRB, 5500 Interstate North Parkway, Suite 515, Atlanta, GA, 30328	BJ8-US10028<br>33 North Dearborn, Chicago, IL, 60602	Site, Central: Aug 04, 2023
Sterling IRB, 5500 Interstate North Parkway, Suite 515, Atlanta, GA, 30328	BJ8-US10029<br>4911 Executive Drive, Peoria, IL, 61614	Site, Central: Aug 07, 2023
Sterling IRB, 5500 Interstate North Parkway, Suite 515, Atlanta, GA, 30328	BJ8-US10030<br>1709 South Rock Road, Wichita, KS, 67207	Site, Central: Jun 21, 2023
Sterling IRB, 5500 Interstate North Parkway, Suite 515, Atlanta, GA, 30328	BJ8-US10031<br>675 Old Ballas Road, Creve Coeur, MO, 63141	Site, Central: Aug 04, 2023
Sterling IRB, 5500 Interstate North Parkway, Suite 515, Atlanta, GA, 30328	BJ8-US10032<br>958C S Kenmore Dr, Evansville, IN, 47714	Site, Central: Aug 08, 2023

EC, ethics committee; IRB, institutional review board.

Table S10. E.ngage Investigators and Study Sites.

Investigator	Primary institution name
**BELGIUM**	
Isabel Leroux-Roels	Center for Vaccinology,
Nikita Hanning	Universiteit Antwerpen - Centrum voor de Evaluatie van Vaccinaties
Muriel Lins	AZ SINT-MAARTEN
**CANADA**	
Naresh Aggarwal	Aggarwal and associates Ltd
Peter Dzongowski	Milestone Research
Gerald Vallieres	Centricity Research- Manna Research - Quebec Location
**POLAND**	
Maria Hlebowicz	PUNKT ZDROWIA Hlebowicz Jakubowski Lekarze sp.p.
Marek Konieczny	Velocity Lublin
Bozena Jachimczak	Pratia MCM Krakow
Marek Dwojak	Synexus Polska Sp z o o Oddzial we Wroclawiu
Malgorzata Sipinska-Surzynska	Synexus Polska Sp. z o.o. Oddzial w Katowicach
Monika Zaczek-Chmielewska	Synexus Polska Sp z o o Oddzial w Warszawie
Milena Kowalewska-Celejewska	Synexus Polska Sp. z o.o. Oddzial w Gdansku
Magdalena Szuflinska-Sidorowicz	MICS Centrum Medyczne Warszawa
Jolanta Augustynowicz-Koziell	MICS Centrum Medyczne Torun
Monika Wrona	Velocity Staszow
Izabela Sein Anand	Synexus Polska Sp. z o.o. Oddzial w Gdynia
**UNITED STATES**	
Harry Studdard	Alliance for Multispeciality Research
Codey Bell	Tekton Research Inc.
Kevin Cannon	Accellacare
Helen Stacey	Diablo Clinical Research, Inc.
Michael Carter	Medical Affiliated Research Center Inc.
Mark Adams	Alliance for Multispeciality Research
Laurence Chu	Benchmark Clinical Research - Austin
Paul Bradley	Velocity Clinical Research
Jorge Caso	Suncoast Research Associates, LLC
Richard Glover	Heartland Research Associates, LLC
Henry Paez	Pharmax Research Clinic Inc
Daniel Johnson	Artemis Institute for Clinical Research
David Fried	Velocity Clinical Research
Antoinette Pragalos	CTI Clinical Trial and Consulting Services
Margaret Rhee	Velocity Clinical Research
Robert Riesenberg	Atlanta Center for Medical Research
William Smith	Alliance for Multispeciality Research
Mark Hutchens	Optimal Research
Murray Kimmel	Optimal Research
Laura Pearlman	Synexus Clinical Research US Inc
Daniel Brune	Optimal Research
Terry Poling	Heartland Research Associates, an AMR Company
Leslie Tharenos	Synexus Clinical Research US Inc
Mohammed Allaw	Synexus Radiant Research, Inc

Table S11. Independent Data Monitoring Committee members.

Expert	Committee
Oliver Cornely	IDMC
Isabel Leroux-Roels	IDMC
Geert Molenberghs	IDMC
Nadia Tornieporth	IDMC
Pierre Van Damme	IDMC

IDMC, Independent Data Monitoring Committee

Figure S1. ExPEC9V O-serotype MOPA antibody response on Day 1 and Day 30 (PPEI) and GMFI (95% CI) from baseline in ExPEC9V O-serotype MOPA-determined functional antibody.

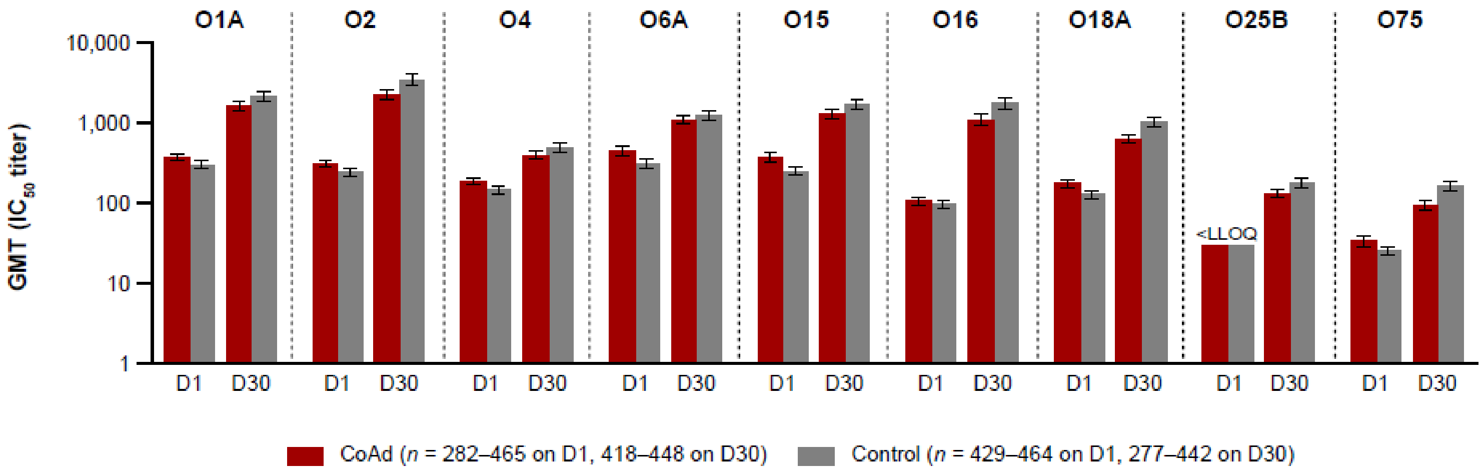

GMFI from baseline (95% CI)									
Serotype	O1A	O2	O4	O6A	O15	O16	O18A	O25B	O75
CoAd	4.40 (3.81–5.09)	7.42 (6.35–8.66)	2.10 (1.87–2.34)	2.36 (2.09–2.66)	3.36 (2.88–3.93)	9.59 (8.29–11.09)	3.81 (3.23–4.49)	2.05 (1.85–2.26)	2.49 (2.11–2.93)
Control	7.18 (6.22–8.29)	13.88 (11.83–16.28)	3.29 (2.91–3.71)	3.67 (3.24–4.15)	6.35 (5.50–7.32)	17.02 (14.71–19.70)	6.80 (5.78–8.00)	2.77 (2.46–3.12)	5.24 (4.49–6.12)

Note: Day is shown relative to ExPEC vaccination. Day 1 O25B data were LLOQ.
LLOQ for O25B was 58. GMFI from pre-vaccination.
Error bars represent the 95% CIs for the GMT. 95% CIs for the GMT and GMFI are based on the t-distribution.
CoAd: ExPEC9V + HD influenza vaccine (Day 1), Placebo (Day 30).
Control: Placebo + HD influenza vaccine (Day 1), ExPEC9V (Day 30).
Abbreviations: CI = confidence interval, CoAd = co-administration, D = Day, ExPEC9V = 9-valent extraintestinal pathogenic *Escherichia coli* vaccine, GMFI = geometric mean fold increase, GMT = geometric mean titer, HD = High-dose, LLOQ = lower limit of quantification, MOPA = multiplex opsonophagocytic assay, PPEI = Per-Protocol ExPEC9V Immunogenicity Set.

Figure S2. ExPEC9V O-serotype multiplex ECL-based immunoassay antibody responses on Day 1 and Day 30 (PPEI) and GMFI (95% CI) from baseline in ExPEC9V O-serotype total (IgG) antibody levels by history of UTI (Control group).

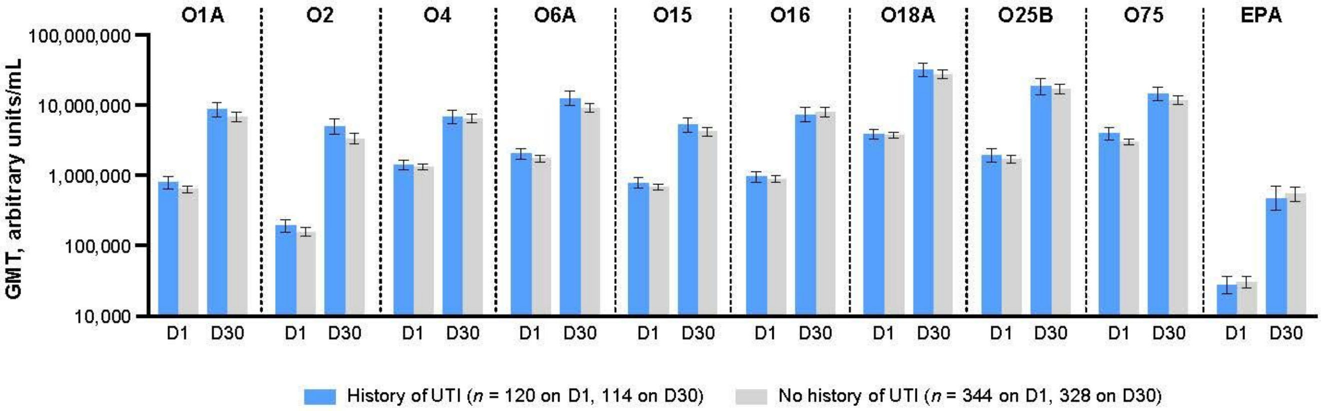

GMFI from baseline (95% CI) – Control Group										
**Serotype**	**O1A**	**O2**	**O4**	**O6A**	**O15**	**O16**	**O18A**	**O25B**	**O75**	**EPA**
History of UTI	10.70 (8.46–13.53)	25.18 (20.31–31.22)	4.73 (3.96–5.64)	6.18 (5.03–7.60)	6.62 (5.30–8.28)	7.82 (6.39–9.57)	8.26 (6.81–10.01)	9.52 (7.34–12.36)	3.69 (3.07–4.43)	15.80 (11.91–20.97)
No history of UTI	10.53 (9.16–12.09)	21.27 (18.19–24.87)	4.86 (4.29–5.49)	5.21 (4.65–5.84)	6.25 (5.52–7.08)	8.85 (7.81–10.04)	7.33 (6.48–8.29)	10.08 (8.67–11.71)	3.96 (3.52–4.46)	15.86 (13.48–18.65)

Note: Day is shown relative to ExPEC vaccination.
GMFI from pre-vaccination.
Error bars represent the 95% CIs for the GMT. 95% CIs for the GMT and GMFI are based on the t-distribution.
CoAd: ExPEC9V + HD influenza vaccine (Day 1), placebo (Day 30).
Control: placebo + HD influenza vaccine (Day 1), ExPEC9V (Day 30).
Abbreviations: CI = confidence interval, CoAd = co-administration, ECL = electrochemiluminescence, D = Day, EPA = ExoProtein A, ExPEC9V = 9-valent extraintestinal pathogenic *Escherichia coli* vaccine, GMFI = geometric mean fold increase, GMT = geometric mean titer, HD = high-dose, PPEI = Per-Protocol ExPEC9V Immunogenicity Set, UTI = urinary tract infection.

Figure S3. ExPEC9V O-serotype MOPA antibody response on Day 1 and Day 30 (PPEI) and GMFI (95% CI) from baseline in ExPEC9V O-serotype MOPA-determined functional antibody by history of UTI (Control group).

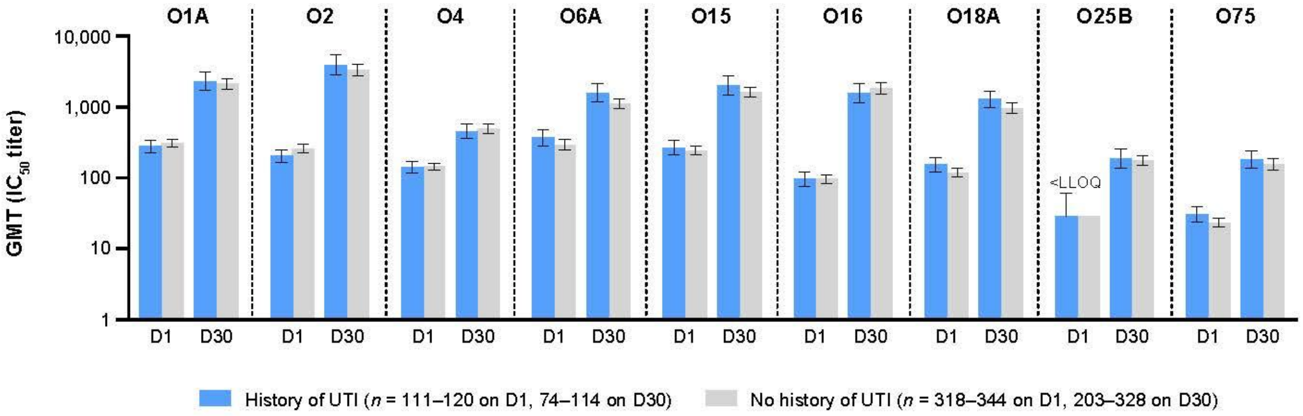

	GMFI from baseline (95% CI) – Control Group								
**Serotype**	O1A	O2	O4	O6A	O15	O16	O18A	O25B	O75
History of UTI	8.38 (6.30–11.16)	18.91 (13.94–25.64)	3.05 (2.44–3.81)	3.89 (2.98–5.09)	6.92 (5.20–9.20)	15.60 (11.83–20.57)	6.75 (4.98–9.15)	2.78 (2.15–3.60)	4.83 (3.55–6.57)
No history of UTI	6.80 (5.76–8.03)	12.45 (10.33–15.01)	3.37 (2.92–3.90)	3.59 (3.12–4.13)	6.16 (5.22–7.27)	17.55 (14.77–20.85)	6.82 (5.61–8.28)	2.77 (2.42–3.16)	5.38 (4.49–6.45)

Note: Day is shown relative to ExPEC vaccination. Day 1 O25B data were LLOQ.
LLOQ for O25B was 58. GMFI from pre-vaccination.
Error bars represent the 95% CIs for the GMT. 95% CIs for the GMT and GMFI are based on the t-distribution.
Control: Placebo + HD influenza vaccine (Day 1), ExPEC9V (Day 30)
Abbreviations: CI = confidence interval, CoAd = co-administration, D = Day, ExPEC9V = 9-valent extraintestinal pathogenic *Escherichia coli* vaccine, GMFI = geometric mean fold increase, GMT = geometric mean titer, HD = high-dose, LLOQ = lower limit of quantification, MOPA = multiplex opsonophagocytic assay, PPEI = Per-Protocol ExPEC9V Immunogenicity Set, UTI = urinary tract infection.
